# Sex differences in serum proteomic profiles in psoriatic arthritis

**DOI:** 10.1093/rheumatology/keaf311

**Published:** 2025-06-25

**Authors:** Steven Dang, Xianwei Li, Liqun Diao, Vincent Piguet, David Croitoru, Joan Wither, Igor Jurisica, Vinod Chandran, Lihi Eder

**Affiliations:** Women’s College Hospital, Toronto, ON, Canada; Institute of Medical Science, University of Toronto, Toronto, ON, Canada; Department of Statistics and Actuarial Science, University of Waterloo, Waterloo, ON, Canada; Department of Statistics and Actuarial Science, University of Waterloo, Waterloo, ON, Canada; Division of Dermatology, Department of Medicine, University of Toronto, Toronto, ON, Canada; Division of Dermatology, Department of Medicine, University of Toronto, Toronto, ON, Canada; Division of Rheumatology, Department of Medicine, University of Toronto, Toronto, ON, Canada; Schroeder Arthritis Institute and Krembil Research Institute, University Health Network, Toronto, ON, Canada; Department of Immunology, University of Toronto, Toronto, ON, Canada; Schroeder Arthritis Institute and Krembil Research Institute, University Health Network, Toronto, ON, Canada; Departments of Medical Biophysics and Computer Science, University of Toronto, Toronto, ON, Canada; Institute of Neuroimmunology, Slovak Academy of Sciences, Bratislava, Slovakia; Institute of Medical Science, University of Toronto, Toronto, ON, Canada; Division of Rheumatology, Department of Medicine, University of Toronto, Toronto, ON, Canada; Schroeder Arthritis Institute and Krembil Research Institute, University Health Network, Toronto, ON, Canada; Department of Laboratory Medicine and Pathobiology, University of Toronto, ON, Canada; Women’s College Hospital, Toronto, ON, Canada; Institute of Medical Science, University of Toronto, Toronto, ON, Canada; Division of Rheumatology, Department of Medicine, University of Toronto, Toronto, ON, Canada

**Keywords:** psoriatic arthritis, proteomics, biomarkers, bioinformatics, women’s health

## Abstract

**Objectives:**

Sex-related differences exist in the clinical presentation and treatment outcomes of patients with PsA. The biological pathways driving these differences remain unknown. We conducted an untargeted proteomic study to identify sex-specific serum proteins and biological pathways in males and females with PsA.

**Methods:**

We used an aptamer-based panel to measure 6402 serum proteins in 50 male and 50 female patients with active PsA and 50 age- and sex-matched non-psoriatic controls. Differential expression and pathway enrichment analysis identified differentially expressed proteins (DEPs) and enriched pathways between male and female PsA patients. Machine learning classifiers were used to develop sex-specific multi-biomarker models to distinguish PsA patients from controls. Proteins with the highest predictive performances were highlighted from random forest models.

**Results:**

The differential analysis revealed over 20 times more sex-specific DEPs in PsA males *vs* controls (*n* = 741) than in PsA females *vs* controls (*n* = 31). The enriched pathways among DEPs in PsA males *vs* PsA females were related to intracellular signalling, vascular function, cytokine signalling and immune cell functions. All models discriminated PsA from controls for both sexes with an area under the curve of 0.85–0.99. Variable importance analysis identified leukotriene A-4 hydrolase as a significant predictor in PsA females *vs* controls, whereas IL-36 alpha, NEK7 and PIK3CA/PIK3R1 were significant in PsA males *vs* controls.

**Conclusion:**

Significantly more dysregulated proteins and biological pathways were found in males than in females with PsA. The identified proteins and pathways offer potential new targets for sex-based research in PsA.

Rheumatology key messages

- PsA males had significantly more deregulated proteins and pathways than females.
- Classification models accurately distinguished PsA from controls for both sexes.
- The identified sex-specific proteins and biological pathways offer new targets for sex-based PsA research.

## Introduction

PsA is an immune-mediated inflammatory condition affecting up to 30% of psoriasis patients [1]. While prevalence is similar in males and females, differences exist in the clinical manifestations and treatment outcomes [2]. Female patients with PsA often present with higher disease activity scores in pain-sensitive domains, including tender joint count, clinical enthesitis and pain scores [2, 3]. They also experience more physical dysfunction, fatigue, work disability and diminished quality of life. Conversely, male patients with PsA tend to have more severe psoriasis, elevated inflammatory markers, and greater radiographic peripheral and spinal joint damage [2, 3]. The mechanisms underlying these differences remain largely unknown. While these mechanisms can be attributed to gender-related socio-cultural differences between men and women (e.g. differences in reporting of pain), sex-related biological mechanisms, particularly differences in immune profiles across sexes, may at least partially explain these differences.

Sex dimorphisms in immune profiles have been well described in healthy individuals with sex hormones affecting the production and function of immune cells, making these changes potentially relevant to PsA [4, 5]. While differences in immune profiles have been reported in axial SpA, our review highlighted the lack of investigation of dimorphisms in the immune pathogenesis of PsA [6–8]. Previous biomarker studies in PsA have not reported sex-disaggregated data, leaving gaps in understanding of whether sex-specific immune-inflammatory profiles influence disease processes [8–10]. A molecular understanding of sex-specific immune mechanisms in PsA is key to moving beyond a simple assessment of differences between males and females and understanding why and how to address them. A sex-specific approach to biomarker discovery could enhance the diagnosis and treatment of PsA and potentially reveal novel targets for sex-specific therapies. In this study, we conducted an untargeted proteomic analysis to identify sex-specific serum proteins and biological pathways in male and female patients with PsA and to develop multi-protein sex-specific classification models that distinguish PsA from controls.

## Methods

### Patients and setting

This cross-sectional study included patients with PsA from the University of Toronto Psoriatic Arthritis Cohort. Patients are examined by a rheumatologist every 6–12 months, with clinical data and blood samples collected following standardized protocols. Blood samples are processed and stored at –80°C in multiple aliquots in our biobank. We searched the cohort database for patients meeting the following criteria: (i) diagnosis of PsA and meeting the ClASsification criteria for Psoriatic ARthritis (CASPAR) criteria [11]; (ii) about to start systemic therapy for active musculoskeletal manifestations of PsA; and (iii) serum samples stored in the biobank. A washout period was observed for patients previously on biologic therapy. We excluded patients with active cancer, end-stage major organ disease or current use of systemic corticosteroids. The study protocol was approved by the Women’s College Hospital Ethics Board (REB # 2023–0010-E). All patients have provided written informed consent at the time of study entry.

### Clinical data collection

The following data on PsA activity were extracted from the cohort database: 68 tender (TJC) and 66 swollen joint counts (SJC), Psoriasis Area and Severity Index (PASI), dactylitis and enthesitis count. US scores for synovitis, tenosynovitis, bone erosions, tendon inflammation, new bone formation, enthesitis inflammation and enthesitis structural damage were also included.

### Proteomic analysis

Serum samples were analysed using the SomaScan 7K assay (SomaLogic, Boulder, CO, USA), a multiplexed, aptamer-based platform that uses slow off-rate modified aptamers (SOMAmer) to bind target proteins [12]. Protein data were standardized and normalized to minimize systematic biases [13]. The assay measured 7289 human protein aptamers, translating to 6402 unique proteins; however, some proteins were targeted by multiple aptamers that recognized distinct epitopes on the same protein. For such cases, we reported the aptamer with the lowest false discovery rate (FDR)-adjusted *P*-value and highest fold-change (FC) to ensure the most biologically relevant measurement.

### Differential expression analysis

The proteomic data were processed using somadataIO (ver.6.1.0) in Rstudio [14]. The data were converted to an expression set, and the relative fluorescence units (RFU) were log2-transformed before being assessed for batch effects. The differential expression analysis was performed using limma (ver 3.58.1), with differentially expressed proteins (DEPs) defined as FDR <0.05 and FC of ≥1.2 [15]. Comparisons included: (i) PsA males *vs* PsA females; (ii) PsA males *vs* control males; and (iii) PsA females *vs* control females.

### Pathway enrichment analysis

We used pathDIP version 5 (https://ophid.utoronto.ca/pathDIP) for pathway enrichment analysis (FDR <0.01) using literature-curated pathways from all 12 database sources and excluding pathways associated with drugs, vitamins and diseases unrelated to PsA [16]. These exclusions ensured a focused and relevant search for sex-specific PsA pathways. We also separated heterodimers into individual entries, and those that met the criteria for differential expression were included in pathway analysis. We focused on enriched pathways differentiating between PsA males and females, manually selecting those relevant to PsA pathology, as determined by domain experts (L.E., V.C.). We also established a minimum threshold of four proteins per pathway to exclude smaller pathways that overlapped with larger ones. The protein-pathway network was created using Network Analysis, Visualization, & Graphing TORonto (NAViGaTOR) version 3 [17].

To reduce redundancy in the pathway enrichment results, we consolidated similar pathways under a common name if they shared >60% of their proteins. For example, three Rho GTPase-related pathways: (i) signalling by Rho GTPases, (ii) signalling by Rho GTPases, Miro GTPases and RHOBTB3, and (ii) RHO GTPase Effectors were grouped under the broader category of ‘Rho GTPase signalling’. Similarly, six angiogenesis-related pathways (i) VEGFA–VEGFR2 signalling, (ii) VEGFR2-mediated cell proliferation, (iii) VEGF signalling pathway, (iv) VEGFA–VEGFR2 pathway, (v) signalling by VEGF, and (vi) angiogenesis were combined under ‘angiogenesis’. This approach allowed us to maintain clarity without losing information.

After noting the absence of expected PsA-related pathways from comparing PsA males *vs* females, we re-evaluated the alignment of our data with the existing PsA literature. We compared the DEPs between PsA males *vs* PsA females (herein referred to as data-driven results) with proteins from the IL-23/17, TNF, IFN-γ and Janus kinase/signal transducer and activator of transcription (JAK–STAT) pathways (literature-driven data) available in pathDIP. Physical protein–protein interactions were assessed using the Integrative Interactions Database [18] version 2021–05 (https://ophid.utoronoto.ca/iid), incorporating data-driven and literature-driven proteins. The interaction network was visualized using NAViGaTOR [17].

### Multi-protein classification model

We developed sex-specific multi-protein classifiers to distinguish PsA from controls using supervised machine learning models. To reduce dimensionality, we identified the union of significant proteins (FDR <0.1) from Student’s *t*-test, Mann–Whitney U test and logistic regression, yielding 4047 proteins for males and 4317 for females. This step reduced the number of variables for modelling while retaining potentially biologically relevant proteins by avoiding overly restrictive *P*-values.

Next, we trained logistic regression with elastic net, linear discriminant analysis, support vector machine and random forest, using the caret package (version 6.0–94) [19]. Each dataset was split into an 80–20 train–test set, with model training conducted via 10-fold cross-validation on the training set and performance evaluated on the test set. The splitting, training and evaluation process was repeated 10 times to calculate each model’s average area under the curve (AUC).

Next, we analysed variable importance from the random forest models using the randomforestSRC package (version 3.3.0) [20]. Random forest was selected for its ability to handle variable interactions and capture nonlinear relationships while providing insights into each variable’s predictive contribution. We adopted a two-stage approach to identify important protein variables for each sex. First, random forest identified variables with importance scores significantly greater than zero (*P* < 0.05). Second, logistic regression with elastic net refined the selected variables. Candidate variables included sex indicators and their interactions with protein variables. This two-stage analysis yielded 44 important protein variables contributing to PsA prediction for each sex. Lastly, we used Spearman rank correlation to examine correlations between these proteins with clinical and US data.

## Results

**Figure 1.**
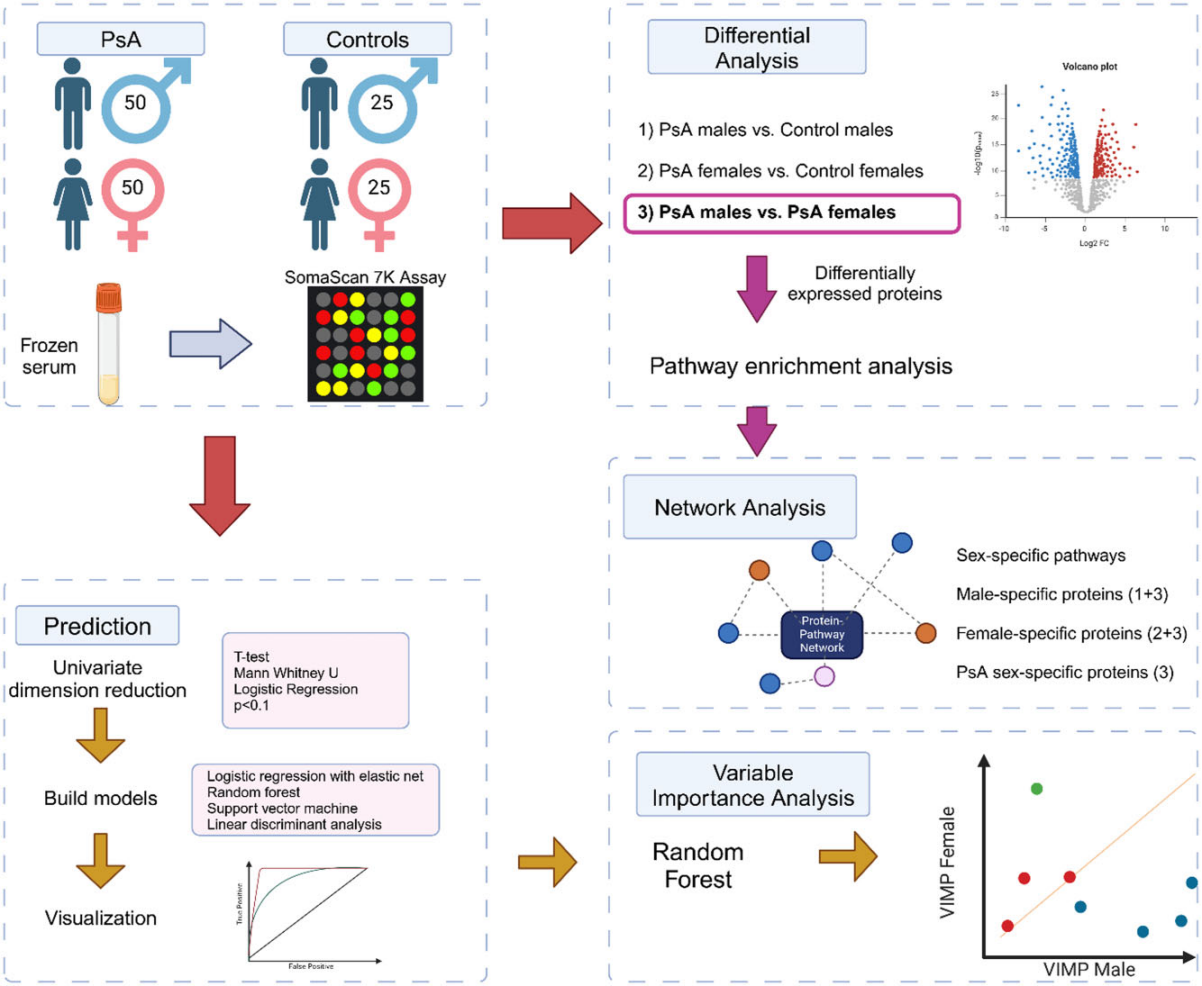
Overview of the study. Starting with 100 active PsA patients and 50 non-psoriatic controls, we measured over 7000 serum protein aptamers. Differential analysis was conducted across three comparison groups. We performed pathway analysis using DEPs from PsA males *vs* PsA females and constructed a network highlighting the interactions. Next, we performed univariate dimension reduction of the proteins to build machine learning models to predict sex-specific disease status (PsA from controls) for each sex. Lastly, we conducted variable importance analysis using the random forest model to identify proteins with significant predictive performances for males and females. Figure was created using BioRender. DEP: differentially expressed protein

We analysed 6402 unique serum proteins in 100 age- and sex-matched patients with active PsA and 50 matched non-psoriatic controls with an equal male-to-female ratio (Fig. 1). The clinical characteristics of PsA patients were similar between males and females (Table 1).

**Table 1.** Patient clinical and demographic characteristics

Variables	PsA		Controls	
	Male (*n* = 50)	Female (*n* = 50)	Male (*n* = 25)	Female (*n* = 25)
Age (years), mean ± s.d.	49.49 ± 12.40	49.40 ± 14.75	47.48 **±** 11.11	46.01 **±** 13.06
BMI (kg/m^2^), mean ± s.d.	29.32 ± 6.24	27.93 ± 7.04		
Smoking, *n* (%)	8 (16)	7 (14)		
PsA disease duration, mean ± s.d.	3.51 ± 5.91	4.46 ± 5.96		
Post-menopause, *n* (%)		21 (42)		
PASI, mean ± s.d.	6.01 ± 6.91	4.56 ± 5.87		
Swollen joint count, median (IQR)	2 ± 6.5	4 ± 5.5		
Tender joint count, median (IQR)	6 ± 6	6 ± 5		
ESR (mm/h), mean ± s.d.	**14.16 ± 17.43**	**24.1 ± 23.15**		
CRP (mg/L), mean ± s.d.	14.91 ± 1.20	15.32 ± 0.96		
bDMARD naïve, *n* (%)	22 (44)	22 (44)		
bDMARD and csDMARD naïve, *n* (%)	12 (24)	15 (30)		

Bolded values indicate statistical differences (*P* < 0.05) based on Mann–Whitney *U* test, χ^2^ or Fisher’s exact test. bDMARDs: biologic DMARDs; csDMARDs: conventional DMARDs; IQR: interquartile range; PASI: Psoriasis Activity and Severity Index.

**Figure 2.**
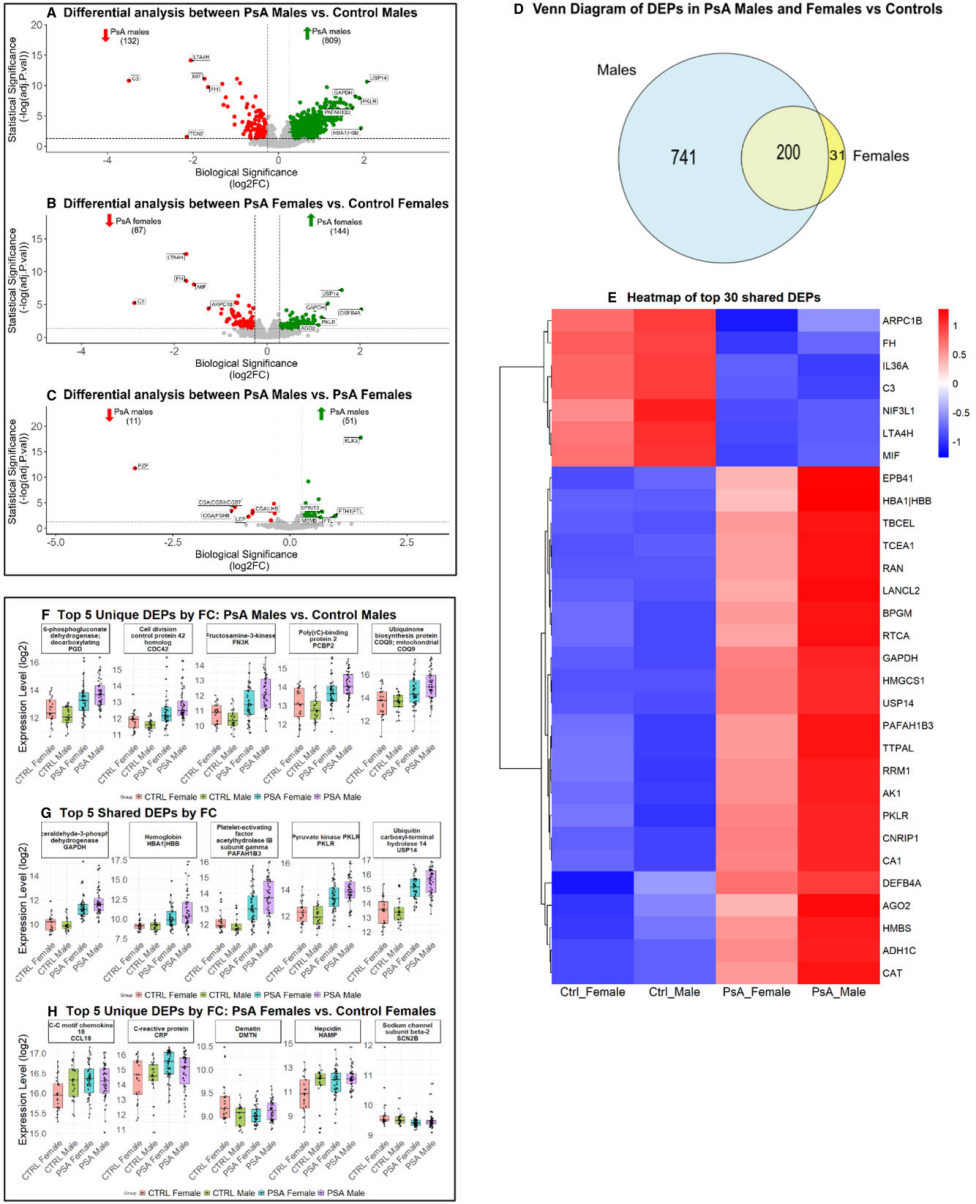
Differential expression analysis was visualized using volcano plots with the top five upregulated and downregulated proteins labelled with the respective Entrez gene symbols for PsA males *vs* control males (**A**); PsA females *vs* Control females (**B**); and PsA males *vs* PsA females (**C**). ; (**D**) Venn diagram of the number of unique sex-specific and shared DEPs between PsA males and females compared with matched controls; (**E**) heatmap of the top 30 shared DEPs by disease and sex; (**F**–**H**) box plots depict the mean fold-change of the top five DEPs in each group: (**F**) male-specific unique DEP; (**G**) shared DEPs; (**H**) female-specific unique DEP. DEP: differentially expressed protein

Differential expression analysis revealed over 20-fold more DEPs in PsA males *vs* control males compared with PsA females *vs* control females (Fig. 2A and B). When comparing PsA males *vs* females, 62 proteins were deregulated (Fig. 2C). Among the deregulated proteins in PsA *vs* controls, 741 were unique to PsA males and 31 in PsA females (Fig. 2D). A heatmap depicting the top 30 shared DEPs by absolute FC shows the same direction of expression in both sexes in PsA and controls, with greater upregulation in PsA males (Fig. 2E) (Supplementary Fig. S1, available at *Rheumatology* online). Sex-based differences in protein expression were largely disease-specific, as only a small subset of DEPs overlapped between PsA males *vs* females and control males *vs* females (7 shared, 3 unique to controls and 56 unique to PsA) (Supplementary Data S1, available at *Rheumatology* online). We also examined the expression levels of the top five unique DEPs by the absolute FC from PsA males *vs* controls (Fig. 2F), PsA females *vs* controls (Fig. 2H) and DEPs shared between the two comparisons (Fig. 2G). Interestingly, protein expression in PsA females *vs* controls (Fig. 2H) tended to cluster more closely between the disease and control groups, potentially suggesting a subtler protein deregulation in females. Detailed differential analysis and pathway results are available in Supplementary Data S1 and S2 and Figs S2–S4, available at *Rheumatology* online.

Proteomic signals (mean RFU) were similar between pre- and post-menopausal PsA females (*P* = 0.77) (Supplementary Fig. S5, available at *Rheumatology* online), but 43 DEPs were identified (Supplementary Table S1, available at *Rheumatology* online). Comparing PsA males with pre- and post-menopausal females revealed 75 and 24 DEPs, respectively, and a statistically significant difference in overall expression between males and pre-menopausal females (*P* = 0.0198), but not post-menopausal females (*P* = 0.095) (Supplementary Figs S6 and S7, available at *Rheumatology* online), suggesting partial convergence following menopause.

**Figure 3.**
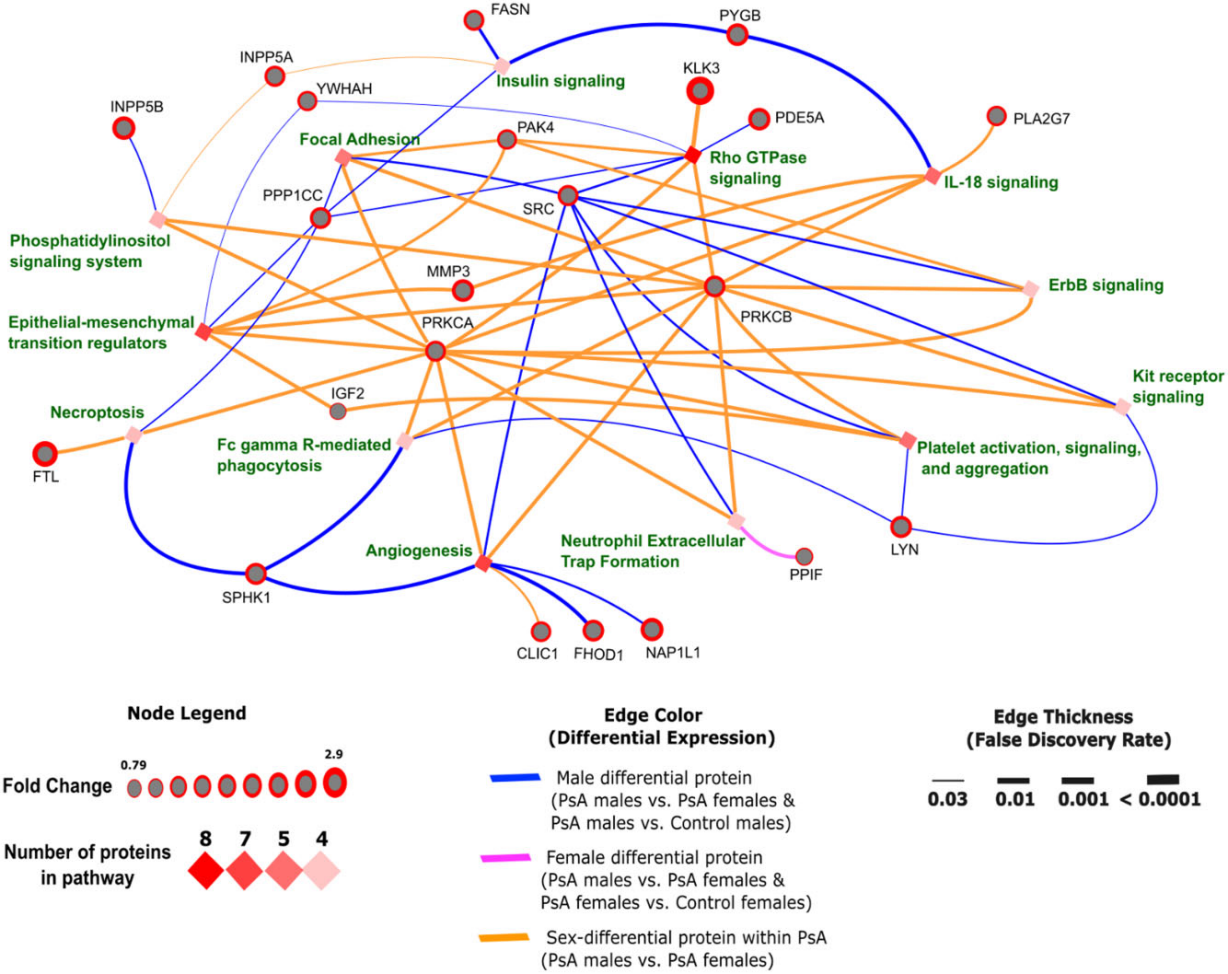
Protein–pathway interactions from DEPs of PsA males *vs* females. Proteins (grey nodes, red highlight for fold-change) connect to pathways (diamond nodes, darker shades for more enriching proteins). Edge colour indicates sex- and disease-specific associations: blue for DEPs in both PsA males *vs* females and PsA males *vs* controls (male differential), pink for PsA males *vs* females and PsA females *vs* controls (female differential) and orange for PsA males *vs* females only (sex-differential within PsA). Edge thickness reflects statistical significance, and thicker protein nodes indicate higher fold changes. DEP: differentially expressed protein

To assess prior treatment effects, we conducted a sensitivity analysis restricted to bDMARD-naïve PsA patients (*n* = 71) (Supplementary Table S2, available at *Rheumatology* online). While DEPs decreased in the PsA *vs* control and PsA male *vs* female comparisons, DEPs increased in PsA males *vs* control males and PsA females *vs* control females. The trend of more DEPs in males persisted, including after adjusting for age. We constructed a network to map interactions between proteins and pathways (Fig. 3). We identified the following male differential proteins (DEP in PsA males *vs* females and PsA males *vs* controls): FASN, PYGB, PDE5A, LYN, FHOD1, NAP1L1, SPHK1, PPP1CC, YWHAH and SRC. Only PPIF was identified as a female differential protein (DEP in PsA males *vs* females and PsA females *vs* controls) (Supplementary Fig. S8, available at *Rheumatology* online). The pathways identified from DEPs between PsA males *vs* females using the criteria described previously included: Rho GTPase signalling, angiogenesis, platelet activation, signalling and aggregation, neutrophil extracellular trap formation (NETosis), insulin signalling, necroptosis, kit receptor signalling, phosphatidylinositol signalling, ErbB signalling, focal adhesion, epithelial–mesenchymal transition (EMT) regulators, IL-18 signalling and Fc gamma R-mediated phagocytosis.

Further, we identified protein–protein interactions between DEPs from PsA males *vs* females (data-driven) and proteins from established PsA-related pathways (literature-driven), which included IL-23/17, TNF, JAK–STAT and IFN-γ. This exercise provided insight into sex differences in a targeted set of proteins known to be implicated in PsA pathogenesis as opposed to an untargeted approach. A substantial number of data-driven proteins were shared across the four PsA-related pathways and interacted extensively with literature-driven proteins (Supplementary Figs S9–S13, available at *Rheumatology* online). Among them, SRC, LYN, PRKCA, PRKCB, YWHAH and several others were present in all four pathways, highlighting their potential central role in PsA pathogenesis. Many of these proteins were male-specific, which may be important in describing unique mechanistic roles or therapeutic targets in PsA males. The full protein–protein interactions for the pathways are found in Supplementary Data S3, available at *Rheumatology* online.

**Figure 4.**
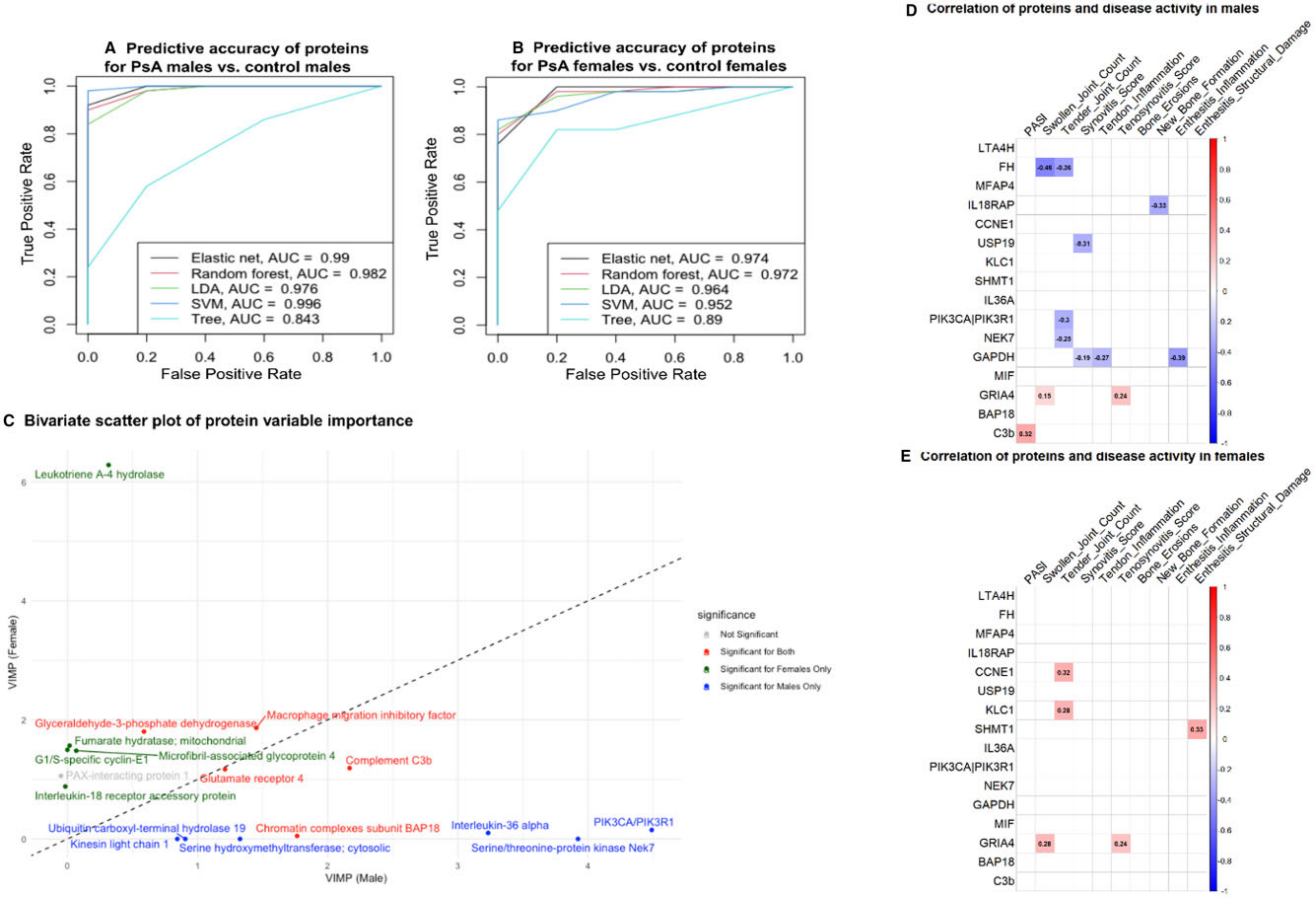
ROC curves for the supervised machine learning models developed using protein data to determine sex-specific disease status (PsA *vs* controls) in (**A**) male PsA *vs* controls and (**B**) female PsA *vs* controls. (**C**) Scatter plot depicting the VIMP of sex-specific proteins included in the random forest model. The *x*-axis of the bivariate scatterplot indicates the importance for males, and the *y*-axis for females, with protein significance colour-coded: blue for male-specific, green for female-specific and red for both. (**D**, **E**) Correlation matrix of sex-specific proteins from the models and clinical and imaging parameters. Statistically significant correlations (*P* < 0.05) are shown. ROC: receiver operating characteristic; DEP: differentially expressed protein; VIMP: variable importance

Next, we assessed the performance of supervised classification models to distinguish PsA from controls within each sex using datasets derived from univariate dimension reduction. Using 4047 proteins for males and 4317 for females, the models demonstrated excellent discriminative ability, with AUC values from 0.85–0.99 (Fig. 4A). Variable importance analysis from random forest identified LTA4H as a key contributor to the predictive accuracy in distinguishing PsA females from controls, whereas IL-36A, NEK7 and PIK3CA/PIK3R1 were key predictors for PsA males *vs* controls (Fig. 4B).

Some proteins, despite being differentially expressed in both sexes, were stronger predictors for one sex than the other, suggesting potential sex-specific disease mechanisms (Supplementary Table S3, available at *Rheumatology* online). Conversely, other proteins with similar expression patterns and differential expression in both sexes remained effective predictors of PsA irrespective of sex (Supplementary Figs S14 and S15, available at *Rheumatology* online).

Taking the list of significant proteins (Fig. 4B), we assessed their correlation with disease activity measures and US scores (Fig. 4C, D). For males, C3b had the strongest correlation with PASI (r = 0.32), and FH had the strongest negative correlation with SJC (r = –0.46). For females, SHMT1 had the strongest positive correlation with US enthesitis structural damage score (r = 0.33).

## Discussion

This untargeted proteomic study explored serum proteins and biological pathways that may contribute to PsA pathogenesis and sex differences. A key finding was the substantially higher number of deregulated proteins in males than in females with PsA. We also identified pathways related to innate immunity (NETosis, phagocytosis), cytokine signalling (IL-18), intracellular signalling (Rho GTPase) and vascular function (angiogenesis, platelet activation). Key male DEPs including SRC, LYN, PPP1CC, YWHAH and SPHK1, and the female-specific PPIF emerged as pivotal contributors. These proteins and pathways are potential sex-specific targets for future biomarker and therapeutic research in PsA.

Our comparative analysis found that males expressed a stark 20-fold more deregulated proteins than females, a pattern also observed in other rheumatic diseases, such as OA, AS and SLE [6, 21, 22]. This disparity may reflect sex-specific immune responses, increased inflammation or greater disease involvement in males. Clinical disease activity scores did not differ significantly between sexes, suggesting that more DEPs in males may not equate to more severe disease. Conversely, fewer but biologically important proteins and pathways in females may exert a comparable impact on PsA severity.

Hormonal fluctuations during the menstrual cycle could contribute to biological variability, potentially masking deregulated proteins. To assess the role of menopause, as a proxy for sex hormone changes, we compared proteomic profiles between pre- and post-menopausal females with PsA and between PsA males and both female subgroups. Our findings suggest that menopause-related changes may partially shift the female proteome toward a male-like profile but do not fully account for the sex-based differences in PsA. The reduced number of DEPs between PsA males and post-menopausal females compared with pre-menopausal females supports this partial convergence. However, differences persisted, which may be due to other factors, such as genetic, epigenetic or chromosomal factors. We also acknowledge the small sample size and lack of hormone data as limitations.

Contrary to expectations, some findings did not align with established PsA literature or showed significant differences from controls. We observed notable differences in the IL-23/17, TNF, IFN-γ and JAK–STAT proteins, and pathways between PsA and controls; however, some proteins exhibited unexpected expressions. These discrepancies may reflect the heterogeneity of PsA, alternative inflammatory pathways or the limitations of serum-based analysis. We addressed this by aligning our data-driven proteins from PsA males *vs* PsA females with established PsA inflammatory pathways. For example, male-specific data-driven proteins like SRC, LYN, YWHAH, NAP1L1, PPP1CC or SPHK1 interacted with several literature-driven proteins across the major pathways. Overall, this contextualizes our results within the broader knowledge of PsA and highlights interactions with literature-driven proteins that may not be readily apparent. Validation is required to confirm whether the proteins may be suitable diagnostic or therapeutic biomarkers.

Dysfunctional angiogenesis and vascular abnormalities are well-established features of PsA pathophysiology [23]. Baggio *et al.* reported stronger angiogenic responses in female donor human umbilical vein endothelial cells, suggesting that endothelial genotype may outweigh the inflammatory microenvironment in driving sex-based differences [24]. In our study, the Rho GTPase signalling pathway, which helps facilitate angiogenic response, exhibited potential sex-related differences. SRC, a central mediator, is a non-receptor tyrosine kinase that influences a myriad of downstream processes, including angiogenesis. The interplay of Rho GTPase and angiogenic pathways highlights their potential effect on sex-related PsA pathology. Interestingly, targeting SRC and members of its family has been explored in treating psoriasis [25–27]. Moreover, Rho GTPase signalling has been shown to predict a favourable response to IL-17A inhibitors in PsA, highlighting its clinical relevance [28].

Another significant pathway in our network was EMT regulators, which involves epithelial cells losing their cell polarity and adhesion, gaining invasive properties. Psoriatic keratinocytes have been shown to exhibit an intermediate type 2 EMT phenotype, involving Erk, Rho and GSK3, which may be driven by upstream IL-17 pathway activation [29, 30]. In our network, male differential proteins YWHAH and PPP1CC bridged EMT and Rho GTPase pathways.

YWHAH (14-3-3 eta) regulates protein trafficking, cell proliferation and signal transduction, and has been studied as a biomarker in inflammatory arthritis [31]. Higher levels of YWHAH were found in the PsA synovial membrane compared with healthy controls and in the blood of patients with erosive PsA [32, 33]. Interactions between the 14-3-3 protein family and Rho proteins may influence Rho GTPase activity, impacting cell cytoskeleton remodelling and cell migration [34].

SPHK1, linked with Fc gamma R–mediated phagocytosis and angiogenesis, was markedly elevated in PsA males. SPHK1 is a bioactive lipid mediator involved in activating TNF and nuclear factor-κB pathways, inducing angiogenesis, and initiating phagosome maturation in macrophages, which activates NLRP3 inflammasomes [35, 36]. In psoriatic keratinocytes, SPHK1 pathway inhibits keratinocyte proliferation, regulates lymphocyte migration and enhances angiogenesis [37]. A study in mice liver models with early fibrosis showed SPHK1 deletion protected female mice from inflammation but not male mice, an effect possibly mediated by oestrogen-mediated crosstalk [38]. This crosstalk has not been investigated in PsA; however, given the role of SPHK1 in our cohort, such research could help clarify sex-specific disease pathways.

PPIF was the only female differential protein in our network associated with NETosis. As a component of the mitochondrial permeability transition pore, PPIF regulates metabolism and reactive oxygen species (ROS) production; dysfunction of this pore can lead to ROS release, cytochrome c leakage and subsequent tissue damage [39]. NETosis, implicated in the pathogenesis of psoriasis and PsA, is suppressed by PPIF in ANCA-associated vasculitis [39–41]. Nonetheless, the mitochondrial metabolic pathways observed from DEPs between PsA females *vs* controls suggest mitochondrial dysfunction as a driver of female-specific disease. Investigating PPIF in NETosis may provide insights into female-specific disease mechanisms in PsA [42].

The classification models showed strong performance in distinguishing PsA males and females from controls, with AUC scores exceeding 0.80. Variable importance analysis identified LTA4H as the top female-specific predictor. Although known for its proinflammatory role in psoriasis, LTA4H levels were decreased in both sexes compared with controls [43]. Interestingly, although CCNE1 was not identified as a sex-specific DEP, it emerged as a key predictor in females, possibly reflecting complex interactions not captured by expression changes alone. While some proteins may contribute to disease pathology in both sexes, their predictive power may be more pronounced for one sex over the other.

Key male-specific proteins included IL-36A, NEK7 and PIK3CA/PIK3R1. IL-36A, a mediator of synovial inflammation, was significantly lower in PsA males compared with controls, warranting the value of examining its levels in SF [44]. NEK7, involved in NLRP3 inflammasome activation, was significantly elevated in PsA males but not in females [45]. PIK3CA/PIK3R1, central to the PI3K–AKT–MTOR pathway, has been linked to the progression from psoriasis to PsA [46]. Our variable importance analysis highlights these proteins as potential drivers of sex-specific PsA pathology and promising candidates for sex-specific biomarker panels.

The variability in protein correlations with disease activity measures may reflect sex-based heterogeneity in PsA mechanisms. A recent study by Eder *et al.* identified PsA sub-phenotypes based on US, suggesting that synovitis, enthesitis and peritenonitis involve distinct inflammatory processes with differential gene activation and unique biological pathways [47]. The differences we observed in relation to SJC with synovitis and TJC with tenosynovitis may be influenced by distinct pathophysiological pathways and sex-specific immune responses. These findings emphasize the need for integrative -omic and imaging studies to better characterize sex-specific mechanisms of inflammation in PsA.

Our study has a few limitations that should be considered. The modest sample size may not be powered to identify all DEPs and enriched pathways, especially given the heterogeneity of PsA. The cross-sectional design also limits our ability to assess the clinical implications of sex-specific protein changes. Nevertheless, to our knowledge, this is the largest study to date examining sex differences in PsA using untargeted proteomics.

An additional limitation is the potential effect of prior biologic therapy on the serum proteome. While most patients were bio-naïve, a washout period based on the drug’s dosing interval was implemented to minimize treatment effects. To assess the influence of prior treatment, we conducted a sensitivity analysis restricted to bDMARD-naïve PsA patients and controls. The overall trends were consistent with the original analysis, with more DEPs in PsA males *vs* controls than in PsA females. However, the increased number of DEPs suggests that prior treatment may have masked some disease-related changes. These findings should be interpreted cautiously due to the smaller sample size and loss of matching. Nonetheless, all patients had active PsA requiring systemic treatment, and including both naïve and exposed individuals improves the real-world relevance of our findings. Future studies in larger bDMARD-naïve cohorts will be important to distinguish disease-specific from treatment-related proteomic changes.

Lastly, the risk of overfitting in our predictive models is a concern due to the high number of protein variables relative to the cohort size. While we performed internal validation and robust testing to mitigate this, further validation in an independent cohort is essential to confirm our findings.

It is worth noting that while we referred to the differences identified in the study as ‘sex differences’, we cannot fully disentangle the potential effect of gender on these changes. Gender-related differences in occupations, lifestyle habits (e.g. smoking and diet) and more could be driving some of the protein differences.

In summary, our proteomic study identified pronounced differences in the number and nature of deregulated biological pathways between male and female patients with PsA. Male patients showed a substantially higher number of deregulated pathways, including inflammatory and immune-related pathways. These findings offer new insights into the sex-specific biological pathways implicated in PsA. This nuanced understanding of sex-specific differences in PsA may lead to more personalized and effective therapeutic interventions tailored to the unique characteristics of male and female patients.

## Supplementary Material

keaf311_Supplementary_Data

## Data Availability

The data that supports the findings of this study are available in the supplementary material of this article.

## References

[1] Ritchlin CT , Colbert RA, Gladman DD. Psoriatic arthritis. New Engl J Med 2017;376:957–70. 28273019 10.1056/NEJMra1505557

[2] Tarannum S , Leung Y-Y, Johnson SR et al Sex- and gender-related differences in psoriatic arthritis. Nat Rev Rheumatol 2022;18:513–26. 35927578 10.1038/s41584-022-00810-7

[3] Coates LC , Van Der Horst-Bruinsma IE, Lubrano E et al Sex-specific differences in patients with psoriatic arthritis: a systematic review. J Rheumatol 2023;50:488–96. 10.3899/jrheum.220386 36243418

[4] Klein SL , Flanagan KL. Sex differences in immune responses. Nat Rev Immunol 2016;16:626–38. 27546235 10.1038/nri.2016.90

[5] Oertelt-Prigione S (2012) The influence of sex and gender on the immune response. Autoimmunity Reviews 11: A479–A485 22155201 10.1016/j.autrev.2011.11.022

[6] Gracey E , Yao Y, Green B et al Sexual dimorphism in the Th17 signature of ankylosing spondylitis. Arthritis Rheumatol 2016;68:679–89. 26473967 10.1002/art.39464

[7] Rusman T , van Vollenhoven RF, van der Horst-Bruinsma IE. Gender differences in axial spondyloarthritis: women are not so lucky. Curr Rheumatol Rep 2018;20:35. 29754330 10.1007/s11926-018-0744-2 PMC5949138

[8] Dang S , Wither J, Jurisica I, Chandran V, Eder L. Sex differences in biomarkers and biologic mechanisms in psoriatic diseases and spondyloarthritis. J Autoimmun 2025;152:103394. 10.1016/j.jaut.2025.103394 40031403

[9] Wirth T , Balandraud N, Boyer L, Lafforgue P, Pham T. Biomarkers in psoriatic arthritis: a meta-analysis and systematic review. Front Immunol 2022;13:1054539. 10.3389/fimmu.2022.1054539 PMC9749424 36532039

[10] Magee C , Jethwa H, FitzGerald OM, Jadon DR. Biomarkers predictive of treatment response in psoriasis and psoriatic arthritis: a systematic review. Ther Adv Musculoskelet Dis 2021;13:1759720X211014010. 10.1177/1759720X211014010 PMC8111521 33995606

[11] Taylor W , Gladman D, Helliwell P et al; CASPAR Study Group. Classification criteria for psoriatic arthritis: development of new criteria from a large international study. Arthritis & Rheumatism 2006;54:2665–73. 16871531 10.1002/art.21972

[12] Gold L , Ayers D, Bertino J, et al Aptamer-based multiplexed proteomic technology for biomarker discovery. PLoS ONE 2010;5:e15004 21165148 10.1371/journal.pone.0015004 PMC3000457

[13] Candia J , Daya GN, Tanaka T, Ferrucci L, Walker KA. Assessment of variability in the plasma 7k SomaScan proteomics assay. Sci Rep 2022;12:17147. 36229504 10.1038/s41598-022-22116-0 PMC9561184

[14] Field S. SomaDataIO: Input/Output 'SomaScan' Data. R package version 6.1.0. 2024. https://somalogic.com and https://somalogic.github.io/SomaDataIO/ (13 December 2024, date last accessed).

[15] Ritchie ME , Phipson B, Wu D et al. Limma powers differential expression analyses for RNA-sequencing and microarray studies. Nucleic Acids Res 2015;43:e47 25605792 10.1093/nar/gkv007 PMC4402510

[16] Pastrello C , Kotlyar M, Abovsky M, Lu R, Jurisica I. PathDIP 5: improving coverage and making enrichment analysis more biologically meaningful. Nucleic Acids Res 2024;52:D663–71 37994706 10.1093/nar/gkad1027 PMC10767947

[17] Brown KR , Otasek D, Ali M et al NAViGaTOR: network analysis, visualization and graphing Toronto. Bioinformatics 2009;25:3327–9. 10.1093/bioinformatics/btp595 PMC2788933 19837718

[18] Kotlyar M , Pastrello C, Ahmed Z, Chee J, Varyova Z, Jurisica I. IID 2021: towards context-specific protein interaction analyses by increased coverage, enhanced annotation and enrichment analysis. Nucleic Acids Res 2022;50:D640–7 34755877 10.1093/nar/gkab1034 PMC8728267

[19] Kuhn M. Building predictive models in R using the caret package. J Stat Softw 2008;28:1–26.

[20] Ishwaran H , Kogalur UB. Fast Unified Random Forests for Survival, Regression, and Classification (RF-SRC). R package version 3.2.0. 2023. https://cran.r-project.org/web/packages/randomForestSRC/randomForestSRC.pdf (13 December 2024, date last accessed).

[21] Yang Y , You X, Cohen JD et al Sex differences in osteoarthritis pathogenesis: a comprehensive study based on bioinformatics. Med Sci Monit 2020;26:e923331-1. 32255771 10.12659/MSM.923331 PMC7163332

[22] Cai M , Gui L, Huang H et al Proteomic analyses reveal higher levels of neutrophil activation in men than in women with systemic lupus erythematosus. Front Immunol 2022;13:911997. 10.3389/fimmu.2022.911997 PMC9254905 35799787

[23] Veale DJ , Fearon U. The pathogenesis of psoriatic arthritis. Lancet 2018;391:2273–84. 29893226 10.1016/S0140-6736(18)30830-4

[24] Baggio C , Boscaro C, Oliviero F et al Gender differences and pharmacological regulation of angiogenesis induced by synovial fluids in inflammatory arthritis. Biomed Pharmacother 2022;152:113181. 35653890 10.1016/j.biopha.2022.113181

[25] Hong J-B , Wu P-Y, Qin A et al Topical tirbanibulin, a dual Src kinase and tubulin polymerization inhibitor, for the treatment of plaque-type psoriasis: phase I results. Pharmaceutics 2022;14:2159. 10.3390/pharmaceutics14102159 PMC9608911 36297594

[26] Chen H , Lu C, Liu H et al Quercetin ameliorates imiquimod-induced psoriasis-like skin inflammation in mice via the NF-κB pathway. Int Immunopharmacol 2017;48:110–7. 10.1016/j.intimp.2017.04.022 28499194

[27] Sundarrajan S , Nandakumar MP, Prabhu D, Jeyaraman J, Arumugam M. Conformational insights into the inhibitory mechanism of phyto-compounds against Src kinase family members implicated in psoriasis. J Biomol Struct Dyn 2020;38:1398–414. 30963942 10.1080/07391102.2019.1605934

[28] Rahmati S , O'Rielly DD, Li Q et al Rho-GTPase pathways may differentiate treatment response to TNF-alpha and IL-17A inhibitors in psoriatic arthritis. Sci Rep 2020;10:21703. 33303908 10.1038/s41598-020-78866-2 PMC7728744

[29] Man X-Y , Chen X-B, Li W et al Analysis of epithelial–mesenchymal transition markers in psoriatic epidermal keratinocytes. Open Biol 2015;5:150032. 26269426 10.1098/rsob.150032 PMC4554915

[30] Guo D , Li X, Wang J et al Single-cell RNA-seq reveals keratinocyte and fibroblast heterogeneity and their crosstalk via epithelial-mesenchymal transition in psoriasis. Cell Death Dis 2024;15:207–14. 38472183 10.1038/s41419-024-06583-z PMC10933286

[31] Cau Y , Valensin D, Mori M, Draghi S, Botta M. Structure, function, involvement in diseases and targeting of 14-3-3 proteins: an update. Curr Med Chem 2018;25:5–21. 10.2174/0929867324666170426095015 28462702

[32] Dolcino M , Ottria A, Barbieri A et al Gene expression profiling in peripheral blood cells and synovial membranes of patients with psoriatic arthritis. PLoS One 2015;10:e0128262 26086874 10.1371/journal.pone.0128262 PMC4473102

[33] Maksymowych WP , Naides SJ, Bykerk V et al Serum 14-3-3η is a novel marker that complements current serological measurements to enhance detection of patients with rheumatoid arthritis. J Rheumatol 2014;41:2104–13. 25128504 10.3899/jrheum.131446

[34] Brandwein D , Wang Z. Interaction between Rho GTPases and 14-3-3 proteins. Int J Mol Sci 2017;18:2148. 29036929 10.3390/ijms18102148 PMC5666830

[35] Weigert A , Olesch C, Brüne B. Sphingosine-1-phosphate and macrophage biology—how the sphinx tames the big eater. Front Immunol 2019;10:1706. 10.3389/fimmu.2019.01706 PMC6658986 31379883

[36] Syed SN , Weigert A, Brüne B. Sphingosine kinases are involved in macrophage NLRP3 inflammasome transcriptional induction. Int J Mol Sci 2020;21:4733. 32630814 10.3390/ijms21134733 PMC7370080

[37] Zhao Y , Zhang Y, Li J et al Pathogenic sphingosine 1-phosphate pathway in psoriasis: a critical review of its pathogenic significance and potential as a therapeutic target. Lipids Health Dis 2023;22:52. 37072847 10.1186/s12944-023-01813-3 PMC10111724

[38] Montefusco D , Jamil M, Maczis MA et al Sphingosine kinase 1 mediates sexual dimorphism in fibrosis in a mouse model of NASH. Mol Metab 2022;62:101523. 35671973 10.1016/j.molmet.2022.101523 PMC9194589

[39] Elrod JW , Molkentin JD. Physiologic functions of cyclophilin D and the mitochondrial permeability transition pore. Circ J 2013;77:1111–22. 23538482 10.1253/circj.cj-13-0321 PMC6397958

[40] Zhang J , Feng Y, Shi D. NETosis of psoriasis: a critical step in amplifying the inflammatory response. Front Immunol 2024;15:1374934. 39148738 10.3389/fimmu.2024.1374934 PMC11324545

[41] Li B , Li G, Yang X et al NETosis in psoriatic arthritis: serum MPO–DNA complex level correlates with its disease activity. Front Immunol 2022;13:911347. 10.3389/fimmu.2022.911347 PMC9238436 35774788

[42] Vorobjeva N , Galkin I, Pletjushkina O et al Mitochondrial permeability transition pore is involved in oxidative burst and NETosis of human neutrophils. Biochim Biophys Acta Mol Basis Dis 2020;1866:165664. 31926265 10.1016/j.bbadis.2020.165664

[43] Iversen L , Kragballe K, Ziboh VA. Significance of leukotriene-A4 hydrolase in the pathogenesis of psoriasis. Skin Pharmacol 1997;10:169–77. 9413890 10.1159/000211501

[44] Boutet M-A , Nerviani A, Lliso-Ribera G et al Interleukin-36 family dysregulation drives joint inflammation and therapy response in psoriatic arthritis. Rheumatology (Oxford) 2020;59:828–38. 10.1093/rheumatology/kez358 PMC7188345 31504934

[45] He Y , Zeng MY, Yang D, Motro B, Núñez G. NEK7 is an essential mediator of NLRP3 activation downstream of potassium efflux. Nature 2016;530:354–7. 10.1038/nature16959 PMC4810788 26814970

[46] Cruz-Correa OF , Pollock RA, Machhar R, Gladman DD. Prediction of Psoriatic Arthritis in Patients With Psoriasis Using DNA Methylation Profiles. Arthritis Rheumatol 2023;75:2178–84. 10.1002/art.42654 37463128

[47] Eder L, , Li Q, , Rahmati S et al Defining imaging sub-phenotypes of psoriatic arthritis: integrative analysis of imaging data and gene expression in a PsA patient cohort. Rheumatology (Oxford, England) 2022;61:4952–61. 10.1093/rheumatology/keac078 PMC9707284 35157043

