# Supplementary material for: Sex differences in serum proteomic profiles in psoriatic arthritis
Source: Rheumatology (Oxford). 2025 Jun 25;64(11):5911–20. doi: 10.1093/rheumatology/keaf311 (PMC12596077; doi:10.1093/rheumatology/keaf311)
Supplement: keaf311_Supplementary_Data [file keaf311_supplementary_data.zip › keaf311_Supplementary_Data/rhe-24-2917-File002.docx]

**Table of Contents:**

**Supplementary Figure S1:** Heatmap of 200 shared proteins

**Supplementary Figure S2:** Dotplot of top 10 pathways for PsA Males vs. Control Males by FDR (A) and gene ratio (B)

**Supplementary Figure S3:** Dotplot of top 10 pathways for PsA Females vs Control Females by FDR (A) and gene ratio (B)

**Supplementary Figure S4:** Dotplot of top 10 pathways for PsA Males vs. Control Males by FDR (A) and gene ratio (B)

**Supplementary Figure S5:** Protein expression between pre- and post-menopausal PsA females

**Supplementary Figure S6:** Protein expression between PsA males and pre-menopausal PsA females

**Supplementary Figure S7:** Protein expression between PsA males and post-menopausal PsA females

**Supplementary Figure S8:** Protein expression from the network of PsA Males vs. PsA Females

**Supplementary Figure S9:** Results from protein-protein interactions between data-driven and literature-driven proteins

**Supplementary Figure S10:** Network between data-driven and literature-driven proteins from IFN-γ pathway

**Supplementary Figure S11:** Network between data-driven and literature-driven proteins from IL-23/17 pathway

**Supplementary Figure S12:** Network between data-driven and literature-driven proteins from JAK-STAT pathway

**Supplementary Figure S13:** Network between data-driven and literature-driven proteins from TNF pathway

**Supplementary Figure S14:** Protein expression from variable importance analysis for PsA Males vs. Control Males

**Supplementary Figure S15:** Protein expression from variable importance analysis for PsA Females vs. Control Females

**Supplementary Table S1:** Differential analysis by menopause status and sex

**Supplementary Table S2:** Sensitivity analysis of DMARD-Naïve patients

**Supplementary Table S3:** VIMP proteins and their differential expression

**Supplementary Data S1:** Results from differential expression analysis (excel file)

**Supplementary Data S2:** Results from pathway enrichment analysis (excel file)

**Supplementary Data S3:** Protein-protein interaction data for PsA-related pathways (excel file)


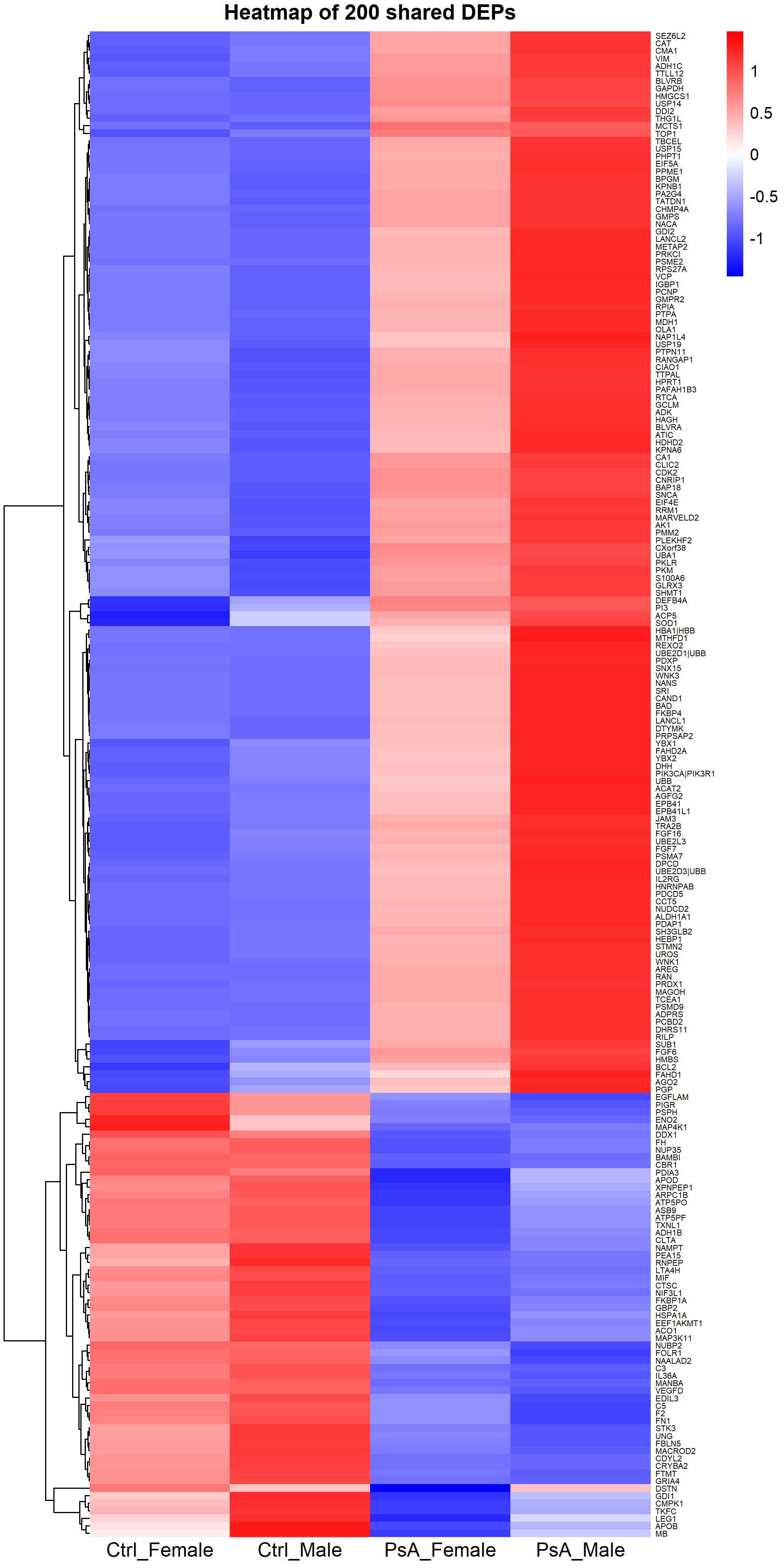


**Supplementary Figure S1.** Heatmap of the 200 differentially expressed proteins shared between PsA males vs. control males and PsA females vs. control females.


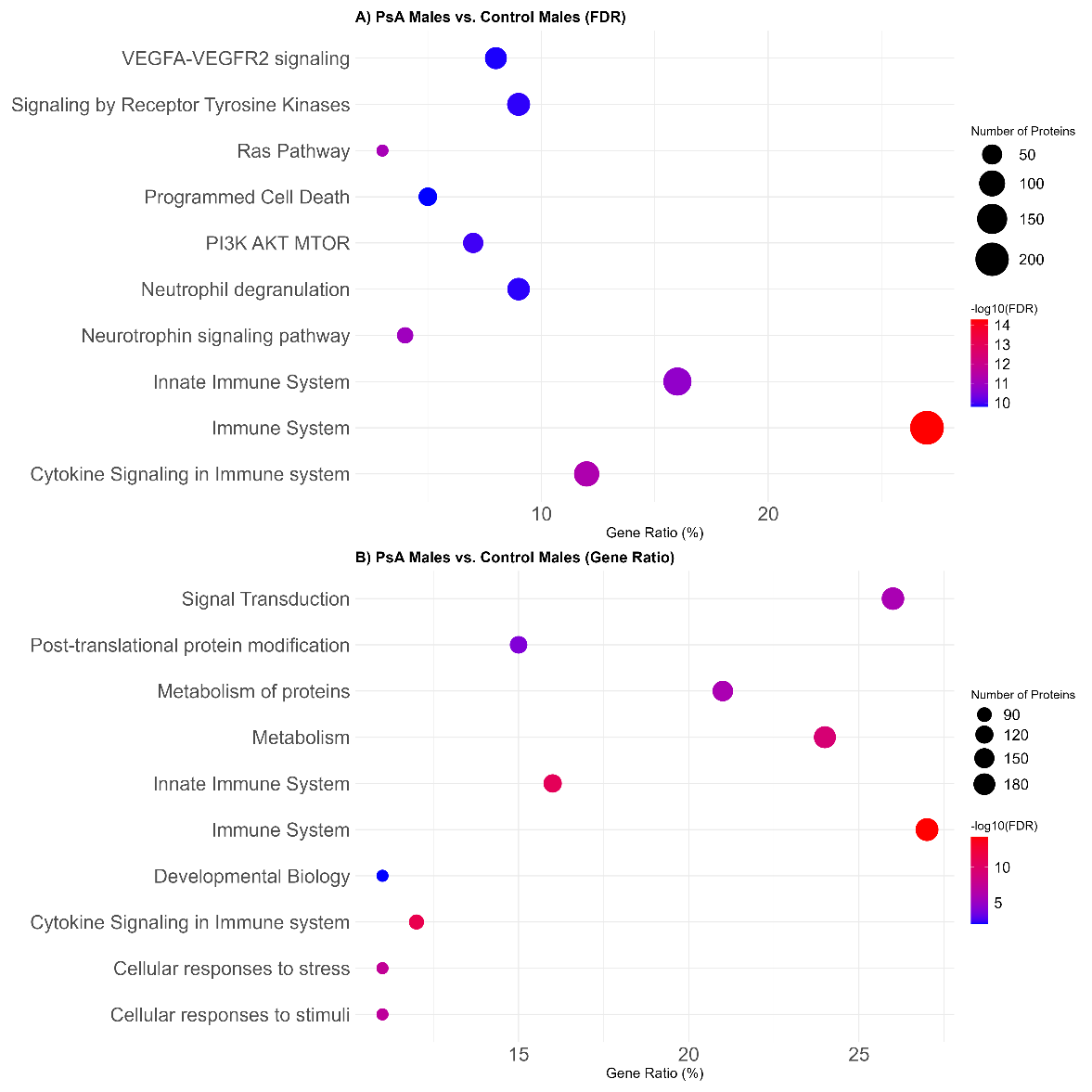


**Supplementary Figure S2.** Dotplot of the top ten pathways from pathway enrichment analysis for DEPs from PsA males vs. control males. (A) Top ten pathways based on lowest FDR. (B) Top ten pathways based on the highest gene ratio


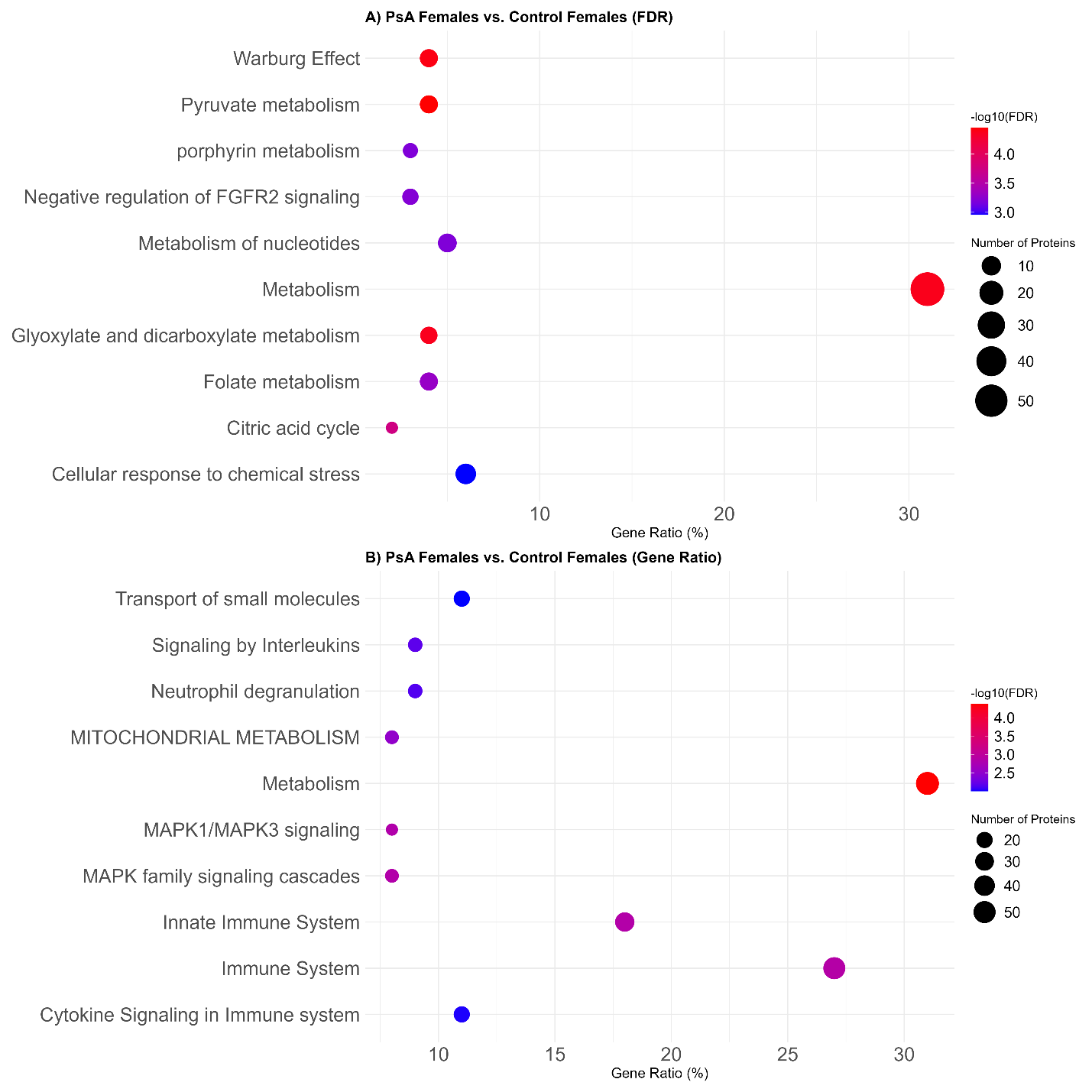


**Supplementary Figure S3.** Dotplot of the top ten pathways from pathway enrichment analysis for DEPs from PsA females vs. control females. (A) Top ten pathways based on lowest FDR. (B) Top ten pathways based on the highest gene ratio

**
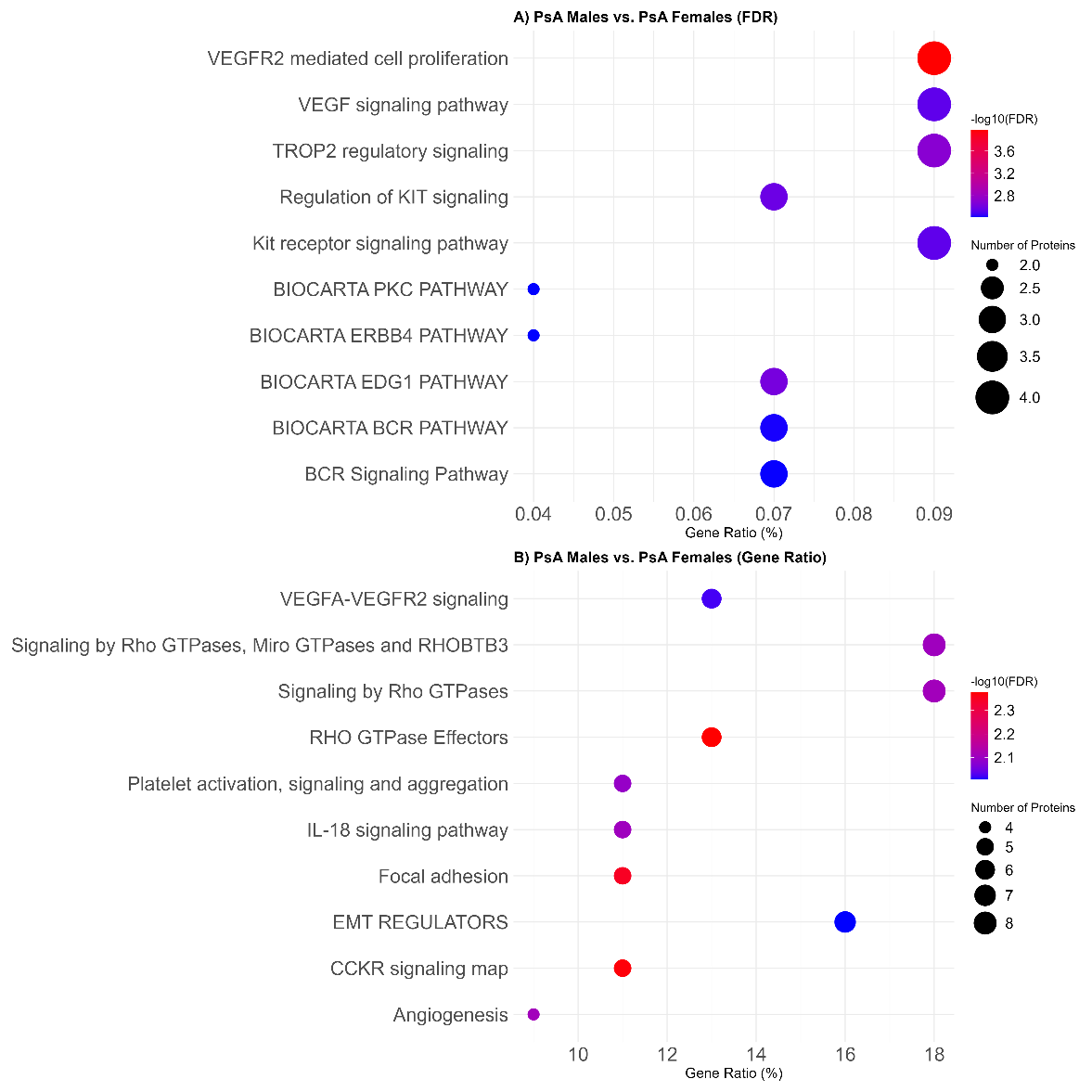
**

**Supplementary Figure S4.** Dotplot of the top ten pathways from pathway enrichment analysis for DEPs from PsA males vs. PsA females. (A) Top ten pathways based on lowest FDR. (B) Top ten pathways based on the highest gene ratio

|  | **Menopausal Status** | |
| --- | --- | --- |
|  | **Pre** | **Post** |
| **Mean Expression** | 11.03 | 11.04 |
| **Standard Deviation** | 0.09178 | 0.1047 |
| **Sample Size** | 29 | 21 |
| **Difference in mean** | -0.008251 | |
| **Pooled SD** | 0.09735 | |
| **DF** | 39.69 | |
| **t-test p value** | 0.7737 | |
| **Levene's test p value** | 0.8107 | |


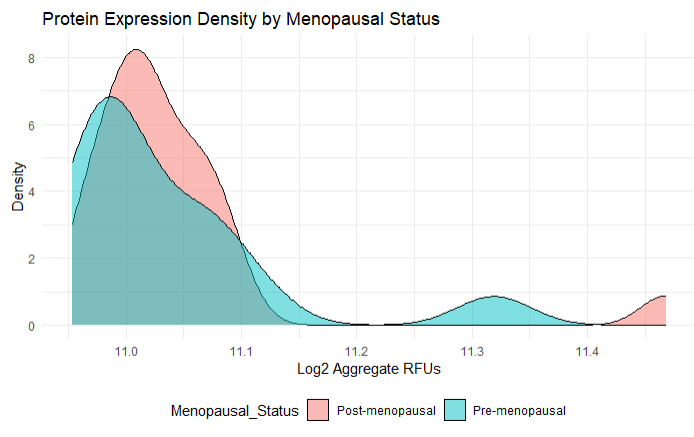


**Supplementary Figure S5.** Protein expression between pre- and post-menopausal PsA females

|  | **Status** | |
| --- | --- | --- |
|  | **PsA Male** | **Pre-menopausal PsA Female** |
| **Mean Expression** | 11.09 | 11.03 |
| **Standard Deviation** | 0.1143 | 0.09178 |
| **Sample Size** | 40 | 29 |
| **Difference in mean** | -0.05602541 | |
| **Pooled SD** | 0.111605 | |
| **DF** | 69.09 | |
| **t-test p value** | 0.01982973 | |
| **Levene's test p value** | 0.1719731 | |


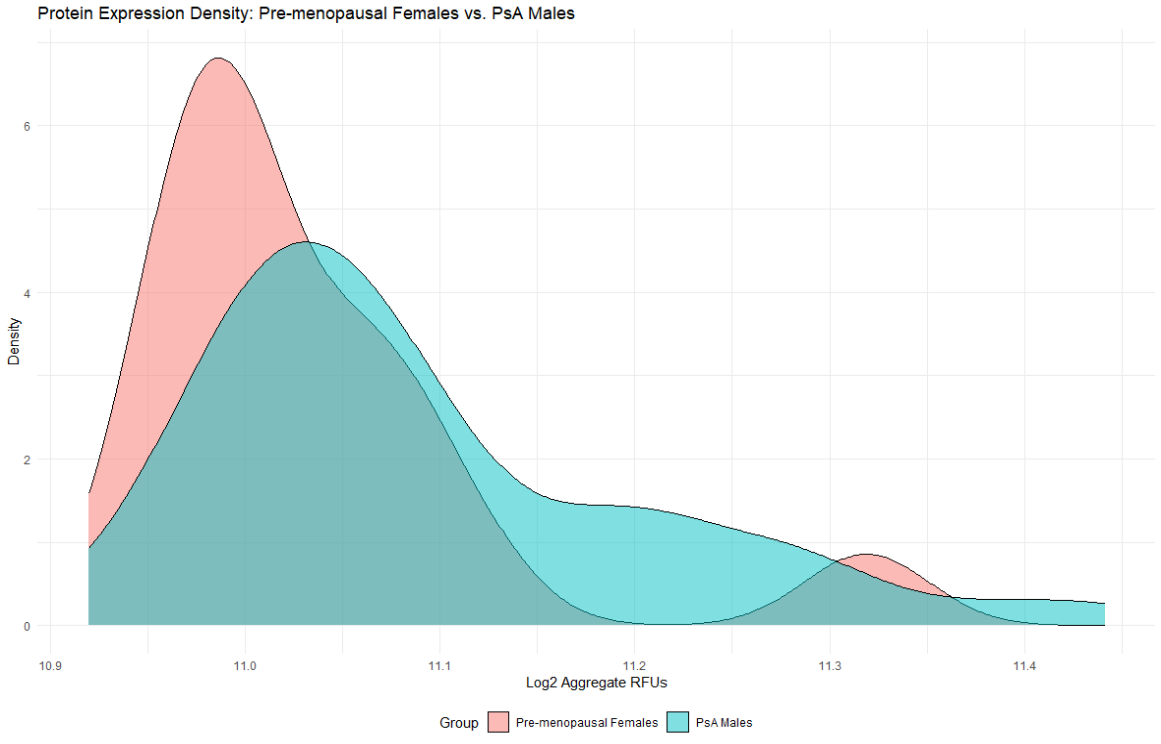


**Supplementary Figure S6.** Protein expression between PsA males and pre-menopausal PsA females

|  | **Status** | |
| --- | --- | --- |
|  | **PsA Male** | **Post-menopausal PsA Female** |
| **Mean Expression** | 11.09 | 11.04 |
| **Standard Deviation** | 0.1143 | 0.1047 |
| **Sample Size** | 50 | 21 |
| **Difference in mean** | -0.04777434 | |
| **Pooled SD** | 0.111605 | |
| **DF** | 40.88 | |
| **t-test p value** | 0.0953492 | |
| **Levene's test p value** | 0.1598 | |


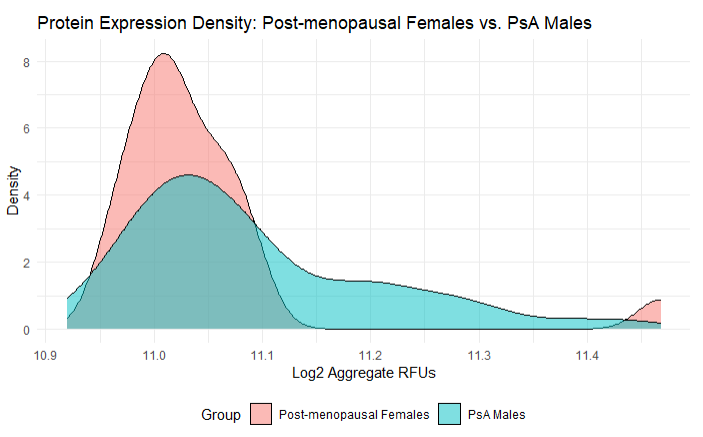


**Supplementary Figure S7.** Protein expression between PsA males and post-menopausal PsA females


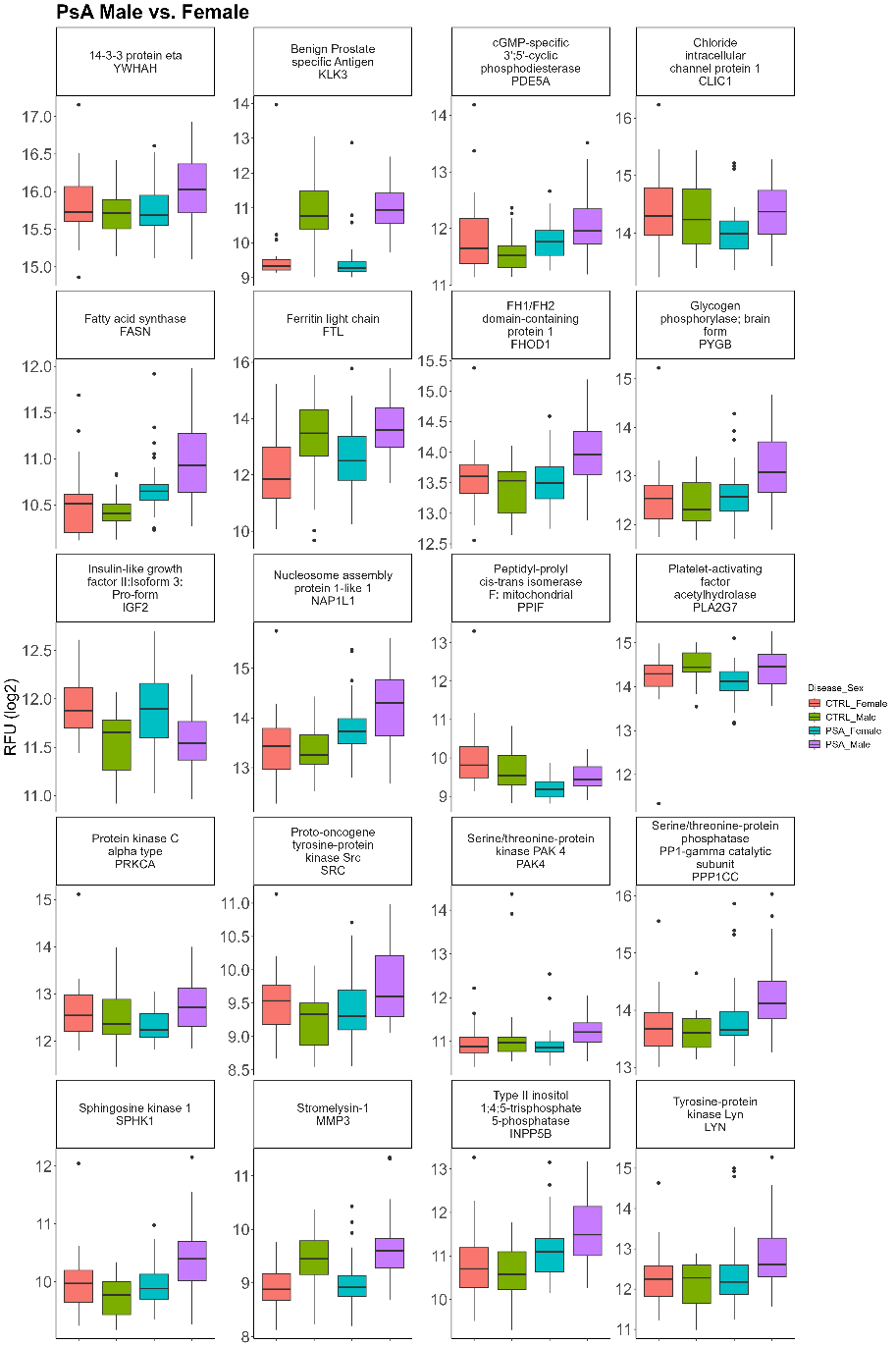


**Supplementary Figure S8.** Boxplots of expression (RFU) of proteins involved in the network of PsA males vs. PsA females for PsA males, PsA females, control males, and control females

**
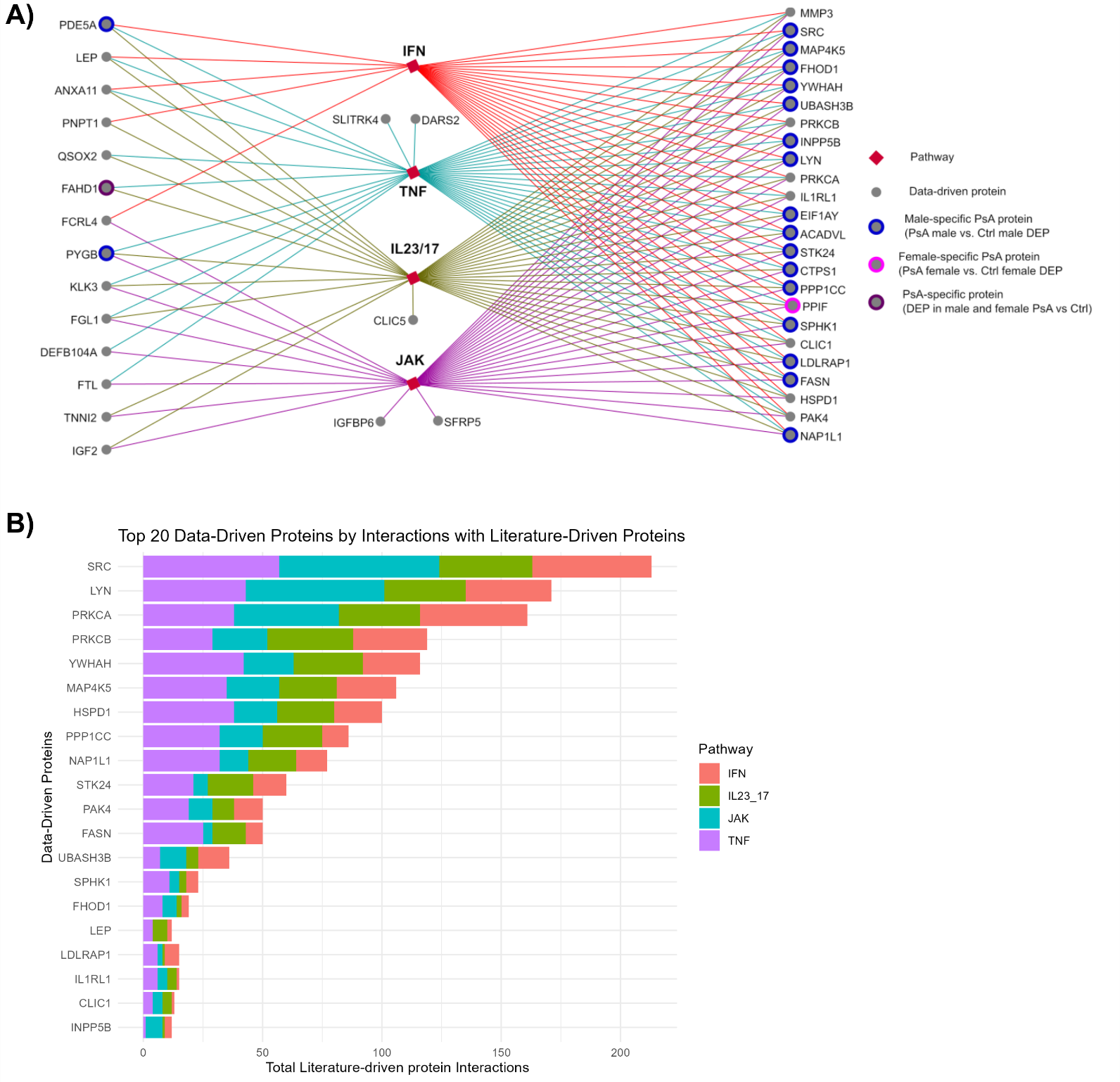
**

**Supplementary Figure S9.** (A) Protein-pathway network of PsA-related pathways (red diamond nodes) and data-driven proteins (differentially expressed in PsA males vs. PsA females) (grey circular nodes). Proteins with blue highlights indicate male-specific PsA protein (also differentially expressed in PsA males vs. control males), pink highlights indicate female-specific PsA protein (also differentially expressed in PsA females vs. control females), and purple highlight indicates PsA-specific protein (differentially expressed in both male and female PsA vs. controls; (B) Stacked bar plot of the PsA-related pathways and the top 20 data-driven proteins by the number of interactions with literature-driven proteins.


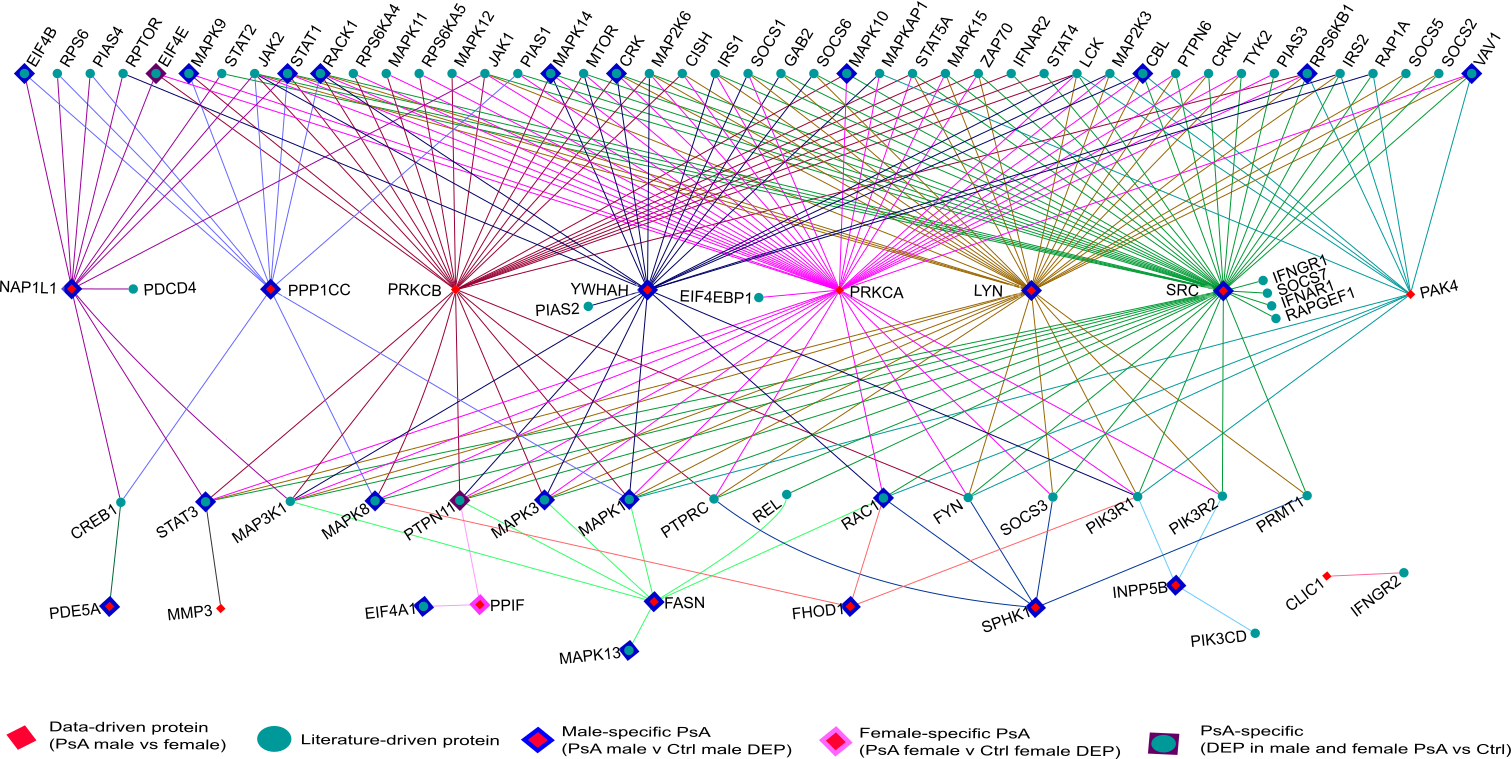


**Supplementary Figure S10.** IFN-γ network illustrating PPI between data-driven proteins (DEPs from PsA males vs. PsA females) and literature-driven proteins from the IFN-γ pathway. Data-driven proteins are represented as red diamond nodes, while literature-driven proteins are shown as turquoise circle nodes. Blue diamond highlights denote proteins also differentially expressed in PsA males vs. control males; pink diamond highlights differential expression in PsA females vs. control females; purple signifies differential expression in both groups. A lack of border around the data-driven proteins indicates its exclusive differential expression in PsA males vs. PsA females.


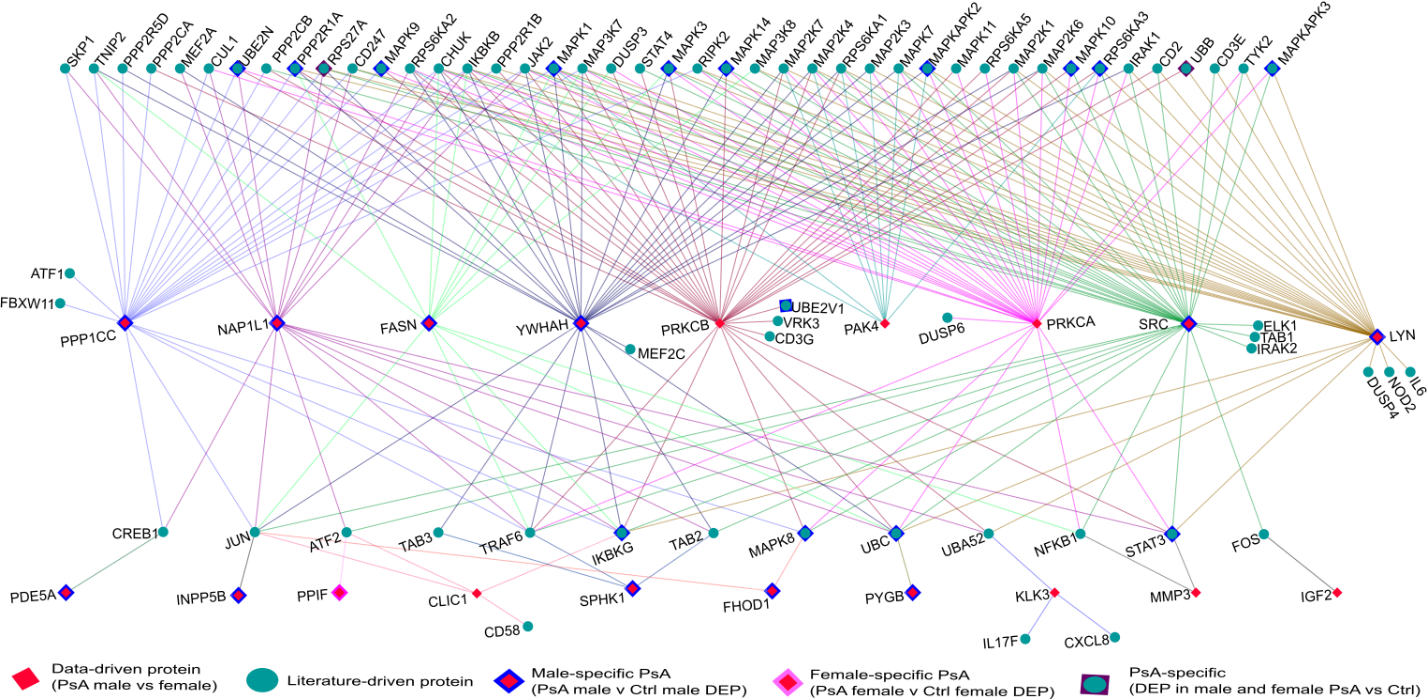


**Supplementary Figure S11.** IL-23/17 network illustrating PPI between data-driven proteins (DEPs from PsA males vs. PsA females) and literature-driven proteins from the IL-23/17 pathway. Data-driven proteins are represented as red diamond nodes, while literature-driven proteins are shown as turquoise circle nodes. Blue diamond highlights denote proteins also differentially expressed in PsA males vs. control males; pink diamond highlights differential expression in PsA females vs. control females; purple signifies differential expression in both groups. A lack of border around the data-driven proteins indicates its exclusive differential expression in PsA males vs. PsA females.


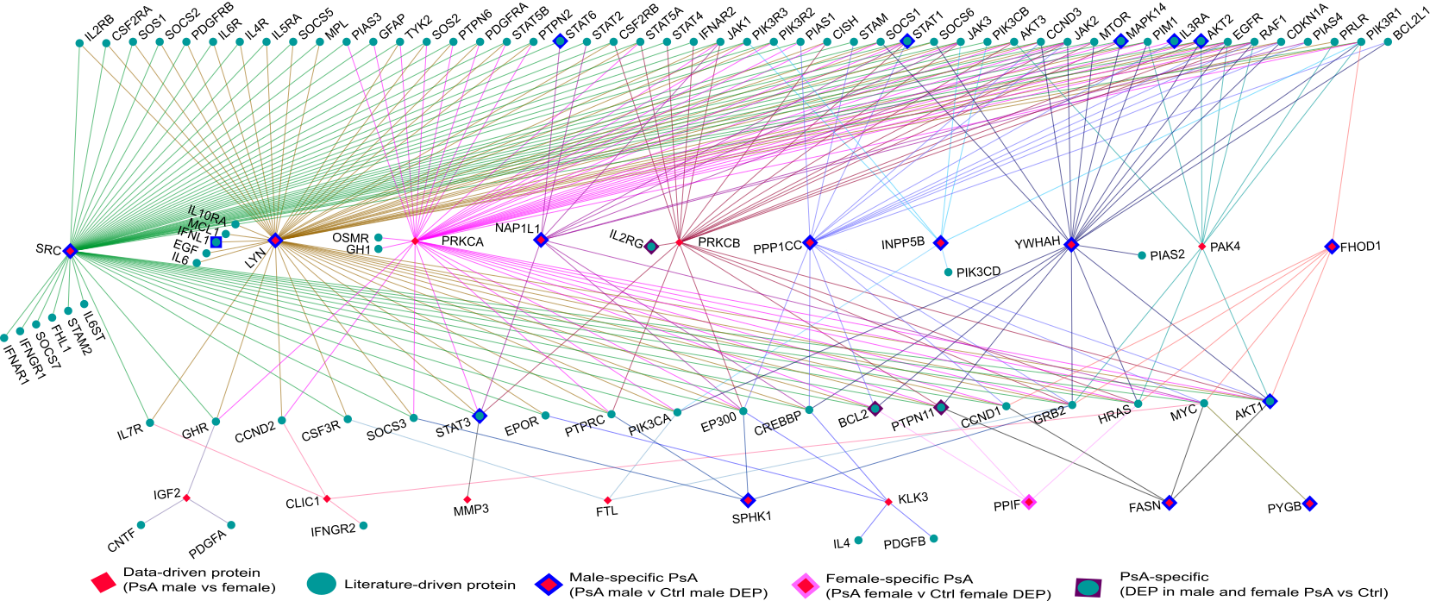


**Supplementary Figure S12.** JAK-STAT network illustrating PPI between data-driven proteins (DEPs from PsA males vs. PsA females) and literature-driven proteins from the JAK-STAT pathway. Data-driven proteins are represented as red diamond nodes, while literature-driven proteins are shown as turquoise circle nodes. Blue diamond highlights denote proteins also differentially expressed in PsA males vs. control males; pink diamond highlights differential expression in PsA females vs. control females; purple signifies differential expression in both groups. A lack of border around the data-driven proteins indicates its exclusive differential expression in PsA males vs. PsA females.


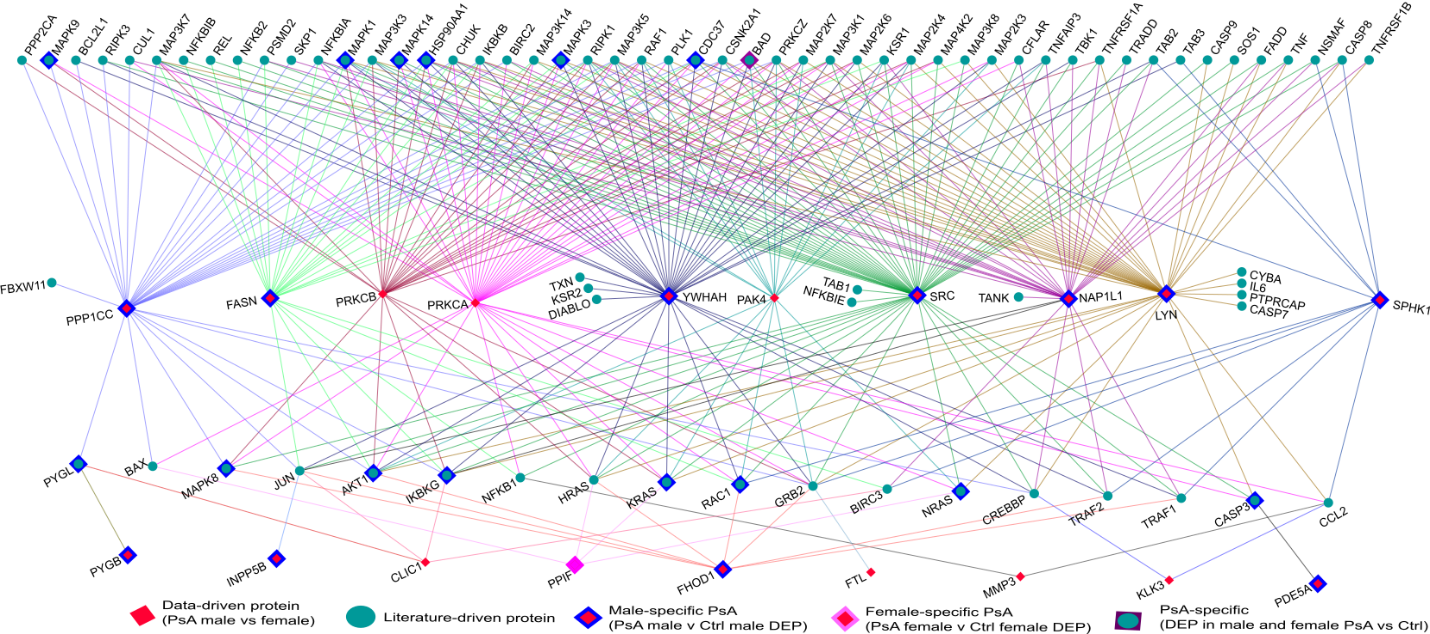


**Supplementary Figure S13.** TNF network illustrating PPI between data-driven proteins (DEPs from PsA males vs. PsA females) and literature-driven proteins from the TNF pathway. Data-driven proteins are represented as red diamond nodes, while literature-driven proteins are shown as turquoise circle nodes. Blue diamond highlights denote proteins also differentially expressed in PsA males vs. control males; pink diamond highlights differential expression in PsA females vs. control females; purple signifies differential expression in both groups. A lack of border around the data-driven proteins indicates its exclusive differential expression in PsA males vs. PsA females.

**
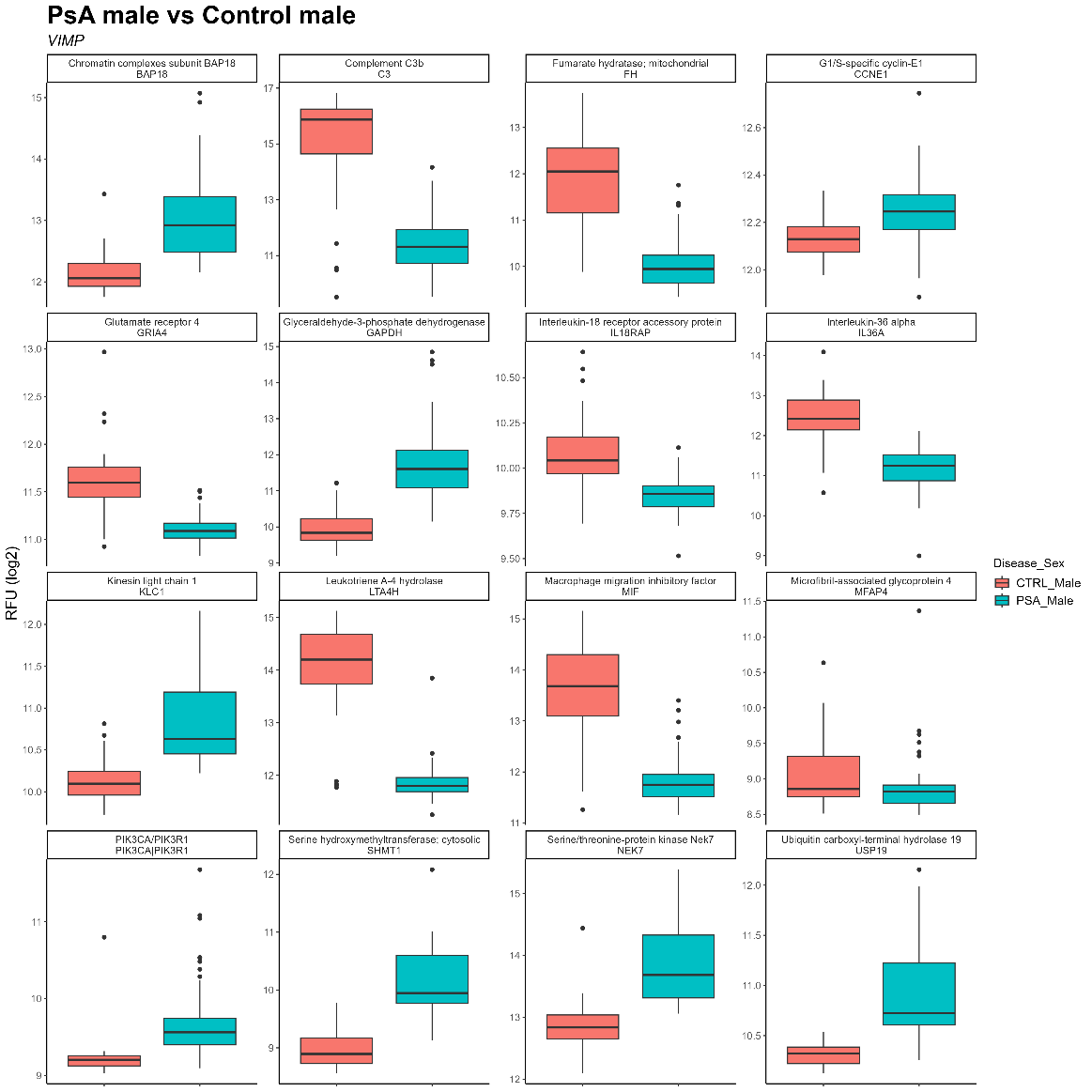
**

**Supplementary Figure S14.** Boxplots of expression (RFU) of proteins identified as important from the variable importance analysis for PsA males vs. control males


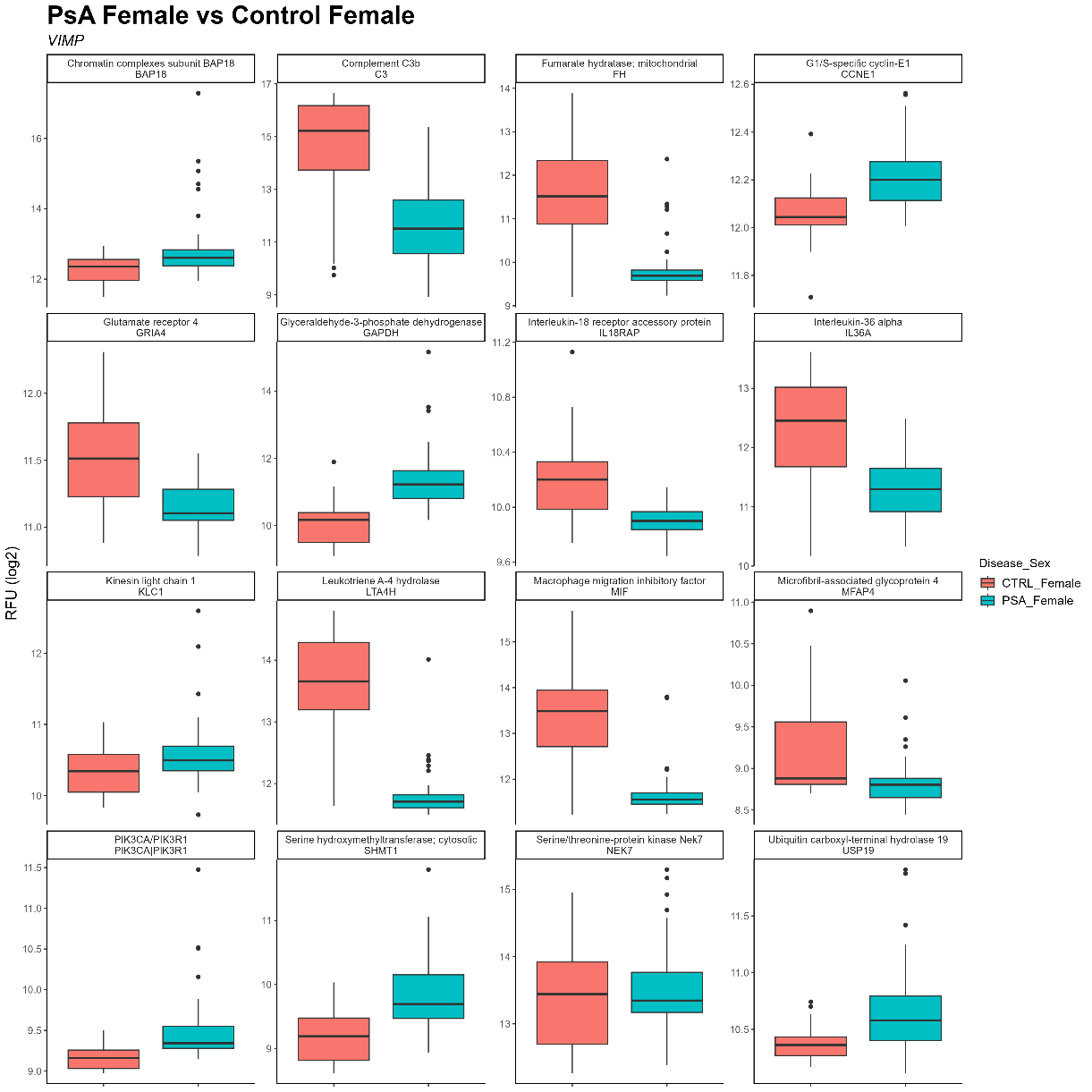


**Supplementary Figure S15.** Boxplots of expression (RFU) of proteins identified as important from the variable importance analysis for PsA females and control females

**Supplementary Table 1.** Differentially expressed proteins by menopausal status and sex in PsA patients

| Comparison |  | **DEPS** | | |
| --- | --- | --- | --- | --- |
|  | **N** | **Upregulated** | **Downregulated** | **Total** |
| Pre- vs. post-menopausal PsA females* | 29/21 | 6 | 37 | 43 |
| PsA males vs. pre-menopausal PsA females* | 50/13 | 62 | 13 | 75 |
| PsA males vs. post-menopausal PsA females* | 50/29 | 12 | 12 | 24 |
| *Comparator |  |  |  |  |

**Supplementary Table 2.** Impact of bDMARD exposure on differential protein expression in PsA patients

| Comparison | **Original** | | | **bDMARD-naïve** | | |
| --- | --- | --- | --- | --- | --- | --- |
|  | **N** | **Upregulated** | **Downregulated** | **N** | **Upregulated** | **Downregulated** |
| PsA vs. Control* | 100/50 | 1612 | 137 | 71/50 | 792 | 181 |
| PsA male vs. Control male* | 50/25 | 809 | 132 | 34/25 | 925 | 202 |
| PsA female vs. Control female* | 50/25 | 144 | 87 | 37/25 | 197 | 90 |
| PsA male vs. PsA female* | 50/50 | 52 | 11 | 34/37 | 17 | 10 |
| *Comparator |  |  |  |  |  |  |

| Protein | PsA male v Ctrl | DEP | PsA female v Ctrl | DEP | VIMP |
| --- | --- | --- | --- | --- | --- |
| BAP18 | Higher | Yes | Higher | Yes | Both |
| C3b | Lower | Yes | Lower | Yes | Both |
| FH | Lower | Yes | Lower | Yes | Female |
| CCNE1 | Higher | **No** | Higher | **No** | Female |
| GRIA4 | Lower | Yes | Lower | Yes | Both |
| GAPDH | Higher | Yes | Higher | Yes | Both |
| IL18RAP | Lower | **No** | Lower | Yes | Female |
| IL36A | Lower | Yes | Lower | Yes | Male |
| KLC1 | Higher | Yes | Higher | **No** | Male |
| LTA4H | Lower | Yes | Lower | Yes | Female |
| MIF | Lower | Yes | Lower | Yes | Both |
| MFAP4 | Lower | **No** | Lower | Yes | Female |
| PIK3CA/PIK3R1 | Higher | Yes | Higher | Yes | Male |
| SHMT1 | Higher | Yes | Higher | Yes | Male |
| NEK7 | Higher | Yes | Lower | **No** | Male |
| USP19 | Higher | Yes | Higher | Yes | Male |

**Supplementary Table 3.** List of proteins identified as important from variable importance analysis, detailing expression levels in PsA vs. controls for both males and females. The table indicates whether each protein’s expression is higher or lower and identifies if it was differentially expressed. Additionally, the table denotes a protein as male-specific, female-specific or significant in both sexes as determined by variable importance analysis
